# Diverse Aquatic Adaptations in *Nothosaurus* spp. (Sauropterygia)—Inferences from Humeral Histology and Microanatomy

**DOI:** 10.1371/journal.pone.0158448

**Published:** 2016-07-08

**Authors:** Nicole Klein, P. Martin Sander, Anna Krahl, Torsten M. Scheyer, Alexandra Houssaye

**Affiliations:** 1 Staatliches Museum für Naturkunde Stuttgart, Rosenstein 1, 70191 Stuttgart, Germany; 2 Steinmann-Institute, Division of Paleontology, University of Bonn, Nußallee 8, 53115 Bonn, Germany; 3 Biomechanics Research Group, Faculty of Mechanical Engineering, Ruhr-Universität Bochum, Universitätsstr. 150, 44801 Bochum, Germany; 4 Palaeontological Institute and Museum, University of Zurich, Karl Schmid-Strasse 4, CH-8006 Zürich, Switzerland; 5 UMR 7179 CNRS/Muséum National d'Histoire Naturelle, Département Ecologie et Gestion de la Biodiversité, 57 rue Cuvier CP-55, 75000 Paris France; Raymond M. Alf Museum of Paleontology, UNITED STATES

## Abstract

Mid-diaphyseal cortical bone tissue in humeri of *Nothosaurus* spp. consists of coarse parallel-fibered bone, finer and higher organized parallel-fibered bone, and lamellar bone. Vascular canals are mainly arranged longitudinally and radially in a dominantly radial system. Blood vessels are represented by simple vascular canals, incompletely lined primary osteons, and fully developed primary osteons. *Nothosaurus* spp. shows a variety of diaphyseal microanatomical patterns, ranging from thick to very thin-walled cortices. In the early Anisian (Lower Muschelkalk), small- and large-bodied *Nothosaurus* spp. generally exhibit bone mass increase (BMI). In the middle to late Anisian (Middle Muschelkalk) small-bodied nothosaurs retain BMI whereas larger-bodied forms tend to show a decrease in bone mass (BMD). During the latest Anisian to early Ladinian (Upper Muschelkalk), small- and few large-bodied nothosaurs retain BMI, whereas the majority of large-bodied forms exhibit BMD. The stratigraphically youngest nothosaurs document five microanatomical categories, two of which are unique among marine amniotes: One consists of a very heterogeneously distributed spongy periosteal organization, the other of very thin-walled cortices. The functional significance of the two unique microanatomical specializations seen in large-bodied nothosaurs is the reduction of bone mass, which minimizes inertia of the limbs, and thus saves energy during locomotion. Transitions between the various microanatomical categories are rather gradual. Our results suggest that small-bodied *Nothosaurus marchicus* and other, not further assignable small-bodied nothosaurs seem to have been bound to near-shore, shallow marine environments throughout their evolution. Some large-bodied *Nothosaurus* spp. followed the same trend but others became more active swimmers and possibly inhabited open marine environments. The variety of microanatomical patterns may be related to taxonomic differences, developmental plasticity, and possibly sexual dimorphism. Humeral microanatomy documents the diversification of nothosaur species into different environments to avoid intraclade competition as well as competition with other marine reptiles. Nothosaur microanatomy indicates that knowledge of processes involved in secondary aquatic adaptation and their interaction are more complex than previously believed.

## Introduction

Sauropterygia was a diverse group of diapsid marine reptiles that existed from the late Early Triassic until the end of the Cretaceous [1,2]. Their Triassic radiation was thought to be restricted to the near-shore habitats of the Tethys Ocean and connected epicontinental seas. It primarily involved shallow marine forms such as Placodontia, Pachypleurosauria, Nothosauroidea, and Pistosauroidea, the latter three clades forming the Eosauropterygia [1]. Nothosauroidea include the well-known genera *Nothosaurus*, *Lariosaurus*, and *Ceresiosaurus*, as well as the only fragmentarily known *Germanosaurus* [1,3]. There is also *Simosaurus*, which is morphologically distinct from the other nothosauroids e.g., [1,4,5].

Alpha taxonomy of nothosaurs is primarily based on skull morphology. The nothosaur skull is dorsoventrally flattened and antero-posteriorly elongated. It has a heterodont dentition, including large fangs in the maxillary bone suggesting piscivory, although stomach contents of *Ceresiosaurus* also include small-bodied marine reptiles [6,7]. Marine invertebrates were also possibly preyed on by nothosaurs, depending on the absolute size of an individual [7]. Postcranial material of nothosaurs is seldom considered to be diagnostic because the postcranial skeletons of eosauropterygians are relatively uniform due to much convergence associated with secondary aquatic adaptations. The most common skeletal elements in classical Muschelkalk bonebed and condensation horizons throughout the Anisian and Ladinian outcrops of the Germanic Basin (Lower to Upper Muschelkalk deposits) [1,8,9,10,11] are isolated bones of *Nothosaurus*. Nothosaur humeri can be distinguish from those of other Sauropterygia due to their dorsoventrally flattened shape, their thin and sharp preaxial margin and the set-off but not constricted midshaft [12]. All nothosaur humeri have an oval to triangular cross section related to the thin and sharp preaxial margin, resembling the cross section of a hydrofoil in some taxa from the Upper Muschelkalk. Morphological details of nothosaur humeri were summarized by Rieppel, [1], Rieppel and Wild [9], Bickelmann and Sander [13], and Klein [12]. Specific taxonomic assignment of isolated *Nothosaurus* humeri is often impossible due to incompletely known association with diagnostic skulls and strong morphological variability, possibly involving sexual dimorphism as well [1,9,14,15]. Large *Nothosaurus*-type humeri also may belong to the large-bodied taxon *Germanosaurus*, which is based on two skulls but lacks associated humeri [1]. On the other hand, *Simosaurus* humeri are distinguishable from those of nothosaurids by their more slender appearance amongst other characters [1,4,5,16].

### Previous studies

Nothosaur skeletons show several morphological aquatic adaptations, such as a dorsoventrally flattened and elongated body and flat and massive girdle bones. *Nothosaurus* exhibits pachyostosis in its ribs, and *Lariosaurus* and *Ceresiosaurus* in humeri, ribs and vertebrae [1]. Braun and Reif [17] interpreted *Nothosaurus* as an anguilliform swimmer. Conversely, muscle reconstructions and morphological observations of the pectoral girdle lead Watson [18], Huene [19], and Carroll and Gaskill [20] to the conclusion that Nothosauroidea had evolved paraxial locomotion. These authors hypothesized that the forelimbs were employed in a “rowing flight”, combining lift- and drag-based elements of propulsion, like in extant sea lions [21]. Contrastingly, the hindlimbs were used for maneuvering. The tail aided to propulsion by undulating movements. The use of the forelimbs for propulsion was recently suggested by trace fossils interpreted as swimming tracks possibly formed by a large nothosaur [22]. Klein et al. [14] hypothesized that *Nothosaurus* from the Lower Muschelkalk used its forelimbs for locomotion, too, based on the dorsoventrally flattened humerus with its oval to triangular cross section.

Klein [12] described a lamellar-zonal bone tissue type with a dominance of longitudinal vascular canals and occasional incipient fibrolamellar bone in nothosaurs from the middle to late Anisian (Middle Muschelkalk). She was able to distinguish two humeral morphotypes in her sample [13]. These morphotypes also differ in histology [12]. In *Ceresiosaurus*, Hugi [23] described lamellar and parallel-fibered bone, as well as a high amount of woven bone with longitudinally and radially arranged vascular canals. Remodelling is limited in *Ceresiosaurus* [23]. The medullary region of *Ceresiosaurus* is filled with calcified cartilage and lamellar bone deposits [23]. She further described and figured two large nothosaur humeri (note that PIMUZ AIII 0001 was incorrectly identified as a femur in Hugi [23]). Hugi [23] pointed out the thin cortex of the humeri, which she interpreted as being related to fast active swimming and preferences for a more open marine environment [23].

Krahl et al. [24] described lamellar-zonal bone tissue in adult *Nothosaurus* and were the first to document differences in microanatomy between small-bodied *N*. *marchicus* from the early Anisian (Lower Muschelkalk) and large-bodied *Nothosaurus* species from the latest Anisian to early Ladinian (Upper Muschelkalk), indicating a shift in locomotion and habitat. Small *N*. *marchicus* was adapted to diving in shallow waters by BMI, whereas the large-bodied species from the Upper Muschelkalk showed an exceptional BMD, suggesting more active swimmers that colonized the open marine environment [24]. Further, the thin-walled, triangular humeral cross sections of large-bodied nothosaurs were interpreted as adaptations for withstanding high bending loads. In agreement with the morphological studies cited above, Krahl et al. [24] hypothesized that the evolution of paraxial front limb propulsion had already taken place in *Nothosaurus*, well before its convergent evolution in the Plesiosauria in the latest Triassic.

*Simosaurus*, the sister taxon of Nothosauridae, grew with lamellar-zonal bone tissue, consisting of coarse parallel-fibered bone tissue with predominantly longitudinal vascular canals. Based on humeral microanatomy, *Simosaurus* was a less efficient (i.e. less active) swimmer than contemporaneous nothosaurs [16,25].

The current study focuses on describing and comparing humeral histology and microanatomy of a large sample of *Nothosaurus* spp. spanning their entire evolutionary history from early Anisian to early Ladinian (Lower to Upper Muschelkalk deposits of the Germanic Basin) in order to document and analyze intrageneric variability. This allows assumptions about the number of possible species occurring in the different stratigraphic horizons and outcrops. Additionally, the study aims at providing insights into aquatic adaptations, detecting evolutionary changes and trends, as well as drawing paleoecological inferences for the genus *Nothosaurus*.

## Material and Methods

### Sampled bones

Sampled humeri from the Lower Muschelkalk (early Anisian) originate from localities in Germany (Eschenbach in der Oberpfalz, Bavaria), Poland (Górny Śląsk), and The Netherlands (Winterswijk). Samples from the Middle Muschelkalk (middle to late Anisian) are from central Germany (Freyburg/River Unstrut, Oberdorla, Rüdersdorf), and those from the Upper Muschelkalk (latest Anisian to early Ladinian) originate from classical Muschelkalk bonebeds of southern Germany [8] (Table 1; S1 Text and S1 Table). All humeri were found in isolation. Additionally, usually only incomplete and damaged bones were available for histological sampling, further limiting morphological information and making specific taxonomic identification difficult. Table 1 lists all specimens sampled together with their collection numbers, locality information, and stratigraphic range. The humeri and their respective thin sections are curated in the following institutions under the collections numbers given in Table 1: IGWH, Institute of Geosciences of the Martin-Luther-University Halle-Wittenberg, Germany; MfN (MB.R.), Museum of Natural History, Leibniz-Institute for Research on Evolution and Biodiversity at the Humboldt University Berlin, Germany; MHI, Muschelkalkmuseum Ingelfingen, Germany; NMNHL RGM (Wijk), National Museum of Natural History Naturalis, Leiden, The Netherlands; PIMUZ, Paleontological Institute and Museum of the University of Zurich, Switzerland; SMNS, Stuttgart State Museum of Natural History, Germany; StIPB, Steinmann-Institute, Division of Paleontology, University of Bonn, Germany; TWE, Museum TwentseWelle, Enschede, The Netherlands. No special permits were required for the described study.

**Table 1:** Measurements (mm) and histological and microanatomical features of *Nothosaurus* spp. samples.

Coll. number	Locality	mw	bl	Medullary area	cc	sl	er	pr	oc	BC	BMI/BMD
**early Anisian (Lower Muschelkalk)**											
SMNS 54317	Eschenbach	5.25	28.4	spongy medullary region	p	p	1	0	sg	89.4	2
SMNS 80154	Eberstadt	5.3	3.1	free cavity partially filled by eb	p	p	1	0	lb	91.4	2
Wijk13-259	Winterswijk	6.2	43*	medullary region filled with cc	p	n	1	0	sg	89.5	2
TWE480000320	Winterswijk	6.3	~35	free cavity	n	n	1	0	sg	82.9	2
Wijk05-9	Winterswijk	9	54*	free cavity	n	n	1–2	0	sg	73.4	2
Wijk11-87	Winterswijk	9.8	54.5	free cavity filled by eb	p	p	1	0	sg	87	2
Wijk13-89	Winterswijk	10.2	63.3	free cavity, perimedullary region	p	p	2	1	lb	92.1	2
MB.R. 782	Górny Śląsk	11.6	> 65	free cavity surrounded by eb	n	n	2	0	lb	93	2
Wijk13-141	Winterswijk	12.3	71.7	free cavity	p	p	1	0	lb	89	2
Wijk11-265	Winterswijk	13.5	73	free cavity	n	n	1–2	0	sg/lb	88.9	2
Wijk10-170	Winterswijk	12.3	78	free cavity, perimedullary region	n	n	1	1	sg	86.4	2
Wijk12-91	Winterswijk	14.5	86.4	free cavity, perimedullary region	n	n	1	1	sg/lb	90.4	2
Wijk11-20	Winterswijk	14.5	95	free cavity, perimedullary region	p	p	2	1	lb	80.8	2
MB.R. 780	Górny Śląsk	42	270*	completely filled by eb	n	n	2	0	lb	93.2	1
MB.R. 817.1	Górny Śląsk	52	320*	spongy medullary region	p	n	1–0	1	lb	70.5	2
**middle to late Anisian (Middle Muschelkalk)**											
MHI 1193	Eberstadt	5.3	3.3	free cavity partially filled by eb	p	p	1	0	sg	88	2
MB.R. 477	Jena	6.6	32.6	medullary region, perimedullary region	p	n	1	1	sg	85.7	2
IGWH 11	Freyburg	6.6	33*	medullary region	n	n	1	1	sg	65.2	2–3
IGWH 12	Freyburg	7.1	33.5*	medullary region	n	n	1	1	sg	59.3	2–3
IGWH 3	Freyburg	10.3	37.3	free cavity	n	n	2	0	sg	82	2–3
**Coll. number**	**Locality**	**mw**	**bl**	**Medullary area**	**cc**	**sl**	**er**	**pr**	**oc**	**BC**	**BMI/BMD**
IGWH 28	Freyburg	16.2	65	free cavity	n	n	0–1	0	sg/lb	68.3	3
IGWH 25	Freyburg	16	76.5	free cavity, perimedullary region	p	n	2	0–1	sg/lb	75.6	3
MB.R. 174.2	Förderstedt	16.3	95*	medullary region, core of cc, perimed. reg.	p	p	1	1	sg	88.9	2
IGWH 14	Freyburg	13	75*	free cavity	n	n	1	0	sg	43.4	4
IGWH 7	Freyburg	15.5	80.5*	free cavity	n	n	0	0	sg	60.2	3
MB.R. 162.4	Oberdorla	11.2	86	free cavity filled by eb	p	n	2	0	lb	91.2	2
IGWH 18	Freyburg	16	94*	free cavity, distally: core of cc	p	p	0	0	lb	70.8	2–3
IGWH 8	Freyburg	20.5	98*	free cavity	n	n	0	0	lb	65.2	3
IGWH 17	Freyburg	21*	110*	free cavity	n	n	0	0	lb	68.4	2–3
MB.R. 414	Freyburg	27.4	145*	free cavity	n	n	0–1	2	lb	71.3	3
IGWH 4	Freyburg	33.5	168*	free cavity, distally: core of cc	p	p	0–1	0	lb	75	2–3
MB.R. 539	Rüdersdorf	21	130	free cavity	n	n	?2	0	lb	74	3
MB.R. 941	Freyburg	41	210	free cavity, perimedullary region	n	n	0–1	1	sg	64.2	2–3
**latest Anisian to early Ladinian (Upper Muschelkalk)**											
GPIT/RE/1339c	unknown	nm	nm	core of cc, erosion cavities	p	p	0	0	sg	nm	na
GPIT/RE/1590d	Crailheim	14	78*	free cavity	n	n	1	0	sg	89.5	2
GPIT/RE/1339b	unknown	15	83*	medullary region with cc	p	n	1	0	sg	89.8	2
GPIT/RE/1590c	Crailsheim	16	67*	core of cc, erosion cavities	p	p	0	0	sg	nm	na
SMNS 53012	Rüblingen	16.6	69.3	free cavity	n	n	1	0	sg	70.6	2
**Coll. number**	**Locality**	**mw**	**bl**	**Medullary area**	**cc**	**sl**	**er**	**pr.**	**oc**	**BC**	**BMI/BMD**
GPIT/RE/1339a	unknown	17	90*	medullary region, redeposition of lb	p	n	0–1	1	sg	83.1	2–3
GPIT/RE/1339f	unknown	19	84	core of cc, erosion cavities	p	p	1	0	lb	92.5	1
GPIT/RE/1590a	unknown	19	85	medullary region, redeposition of lb	p	n	0–1	0	lb	86	2
GPIT/RE/1590b	Crailsheim	19	85*	medullary region, redeposition of lb	n	n	1	0	sg/lb	93.3	1–2
GPIT/RE/1339d	unknown	19	102*	medullary region, redeposition of lb	p	p	1	0	sg	92.7	1
SMNS 2557	Hoheneck	22.8	112.2	medullary region with cc	p	p	1	0	sg	93	1
MHI 1906	Schwb.-Hall	23	53	medullary region	p	n	1	0	sg	86.5	2
MHI 633	Schmalfelden	25	>56	free cavity	n	n	?0	0	sg	84.8	2
MHI 1978	Wilhelmsglück	28.3	183	free cavity	n	n	1	1	sg/lb	89.7	2
SMNS 17214	Crailsheim	29.3	160*	free cavity, few sec. trab.	n	n	0	0–1	sg/lb	58.4	3
SMNS 50221	Rüblingen	30	152.5	medullary region	n	n	1	2	lb	81.1	2–3
StIPB R54/2	Bayreuth	32	200	free cavity, sec. trab.	n	n	0	1–0	lb	53.5	3–4
MHI 754	Satteldorf	33.5	180*	free cavity, perimedullary region	n	n	1	1	lb	72	3
SMNS 84851	Crailsheim	35.4	180*	free cavity, perimedullary region	n	n	2–1	1–0	sg/lb	90.5	2
SMNS 84772	Bindlach III	35.8	165	free cavity, few sec. trab.	n	n	0	2	lb	71.7	3
SMNS 81884	Mistlau	37.2	~200	two large free cavities thinly lined by eb	n	n	0	0	sg	52.4	2–3
SMNS 7175	Hoheneck	38.9	210*	medullary cavity with redeposition of lb	n	n	2–1	0	sg/lb	95.2	1–2
MB.R. 279	Bayreuth	42	236*	free cavity, few sec. trab.	n	n	0	0	sg	47.2	4
MB.R. 282	Bayreuth	42	236*	free cavity, few sec. trab.	n	n	0	2	lb	78.5	3
MB.R. 278	Bayreuth	45	250*	free cavity with remains of eb	n	n	0	1–2	sg/lb	45.3	4
SMNS 81885	Zuffenhausen	46.7	260*	free cavity, few sec. trab.	n	n	0	1–2	lb	32*	3–4
StIPB R 45	Bayreuth	47	>115	free cavity, few sec. trab.	n	n	0	0	sg/lb	50	4
MB.R. 281	Bayreuth	48	270*	free cavity with remains of eb	n	n	0	1	sg	69.3	3–4
PIMUZ AIII-1	Bayreuth	50.5	>225	free cavity, few sec. trab.	n	n	0	1–0	sg	26.1	4
PIMUZ 4845	Ticino	53	232	medullary region	n	n	1–2	0	sg/lb	94.8	1
MHI 873	Schmalfelden	54	290*	free cavity, few sec. trab.	n	n	0	1	sg/lb	38	4
SMNS 80688	Herdlingshaus.	54	290*	free cavity, perimedullary region	n	n	1	1	sg/lb	84.7	2
StIPB R 53	Bayreuth	54	>225	free cavity, few sec. trab.	n	n	0	0	lb	23.3	4
StIPB R 40	Bayreuth	55	>125	free cavity, few sec. trab.	n	n	0	1	sg/lb	34.3	4
MB.R. 270	Bayreuth	55.5	350	free cavity, sec. trab.	n	n	0	1	sg/lb	71.3	2
MB.R. 272	Bayreuth	56	305*	free cavity, few sec. trab.	p	n	0	1–2	sg	37.9	4
SMNS 81988	Gundelsheim	57.5	310*	free cavity, few sec. trab.	n	n	0	1	sg	53	3–4
SMNS 17882	Crailsheim	57.8	320*	free cavity, few sec. trab.	n	n	0	1	lb	46	3–4
PIMUZ AIII-2	Bayreuth	~60	>225	free cavity, sec. trab.	n	n	0	1	lb	36.1	4
MB.R. 269	Ludwigsburg	74	400	large cavities traversed by sec. trab.	n	n	0	1	lb	49.7	4

Abbreviations

*, reconstructed; BC, bone compactness in %; bl, bone length; BMI, bone mass increase; BMD, bone mass decrease [1 = compact bone with reduced medullary cavity (true BMI), 2 = compact, moderately sized cortex and medullary cavity, 3 = enlarged medullary cavity, reduced cortex, 4 = thin-walled humeral cross section (true BMD)]; cc, calcified cartilage; eb, endosteal bone; er, endosteal resorption indicated by lining with endosteal bone [0 = not lined by endosteal bone (active endosteal resorption), 1 = incompletely lined (ongoing endosteal resorption), 2 = completely lined by endosteal bone (endosteal resorption stopped)]; lb, lamellar bone; mw, midshaft width; Mu., Muschelkalk; n, not present; na, not applicable; nm, not measurable; oc, outer cortex; p, present; pr., resorption of periosteal bone [0 = none, 1 = resorption of periosteal bone patchily extending into the mid-cortex, 2 = resorption of periosteal bone reaches into the outer cortex]; sg, still growing; sl, sharp line; vc, vascular canals.

### Taxonomic assignment

Four humeral morphotypes were recognized for nothosaurs; three by Bickelmann and Sander [13] and a fourth by Klein [12]. While these worked well in categorizing the then relatively small sample of Lower to Middle Muschelkalk nothosaur humeri, they lost their distinction in the currently available larger sample. Only humeral morphotype II, erected by Bickelmann and Sander [13], can be linked to a specific taxon, *N*. *marchicus* [14, 13].

Large humeri from the Upper Muschelkalk are often assigned to *N*. *mirabilis* or *N*. *giganteus* [24], although these assignments are not based on skeletal association because no large humerus has been found with an associated skull in the Muschelkalk deposits so far. The humerus of the complete skeleton of *N*. *giganteus*, formerly *Paranothosaurus amsleri*, [26], from the Alpine Triassic is difficult to compare to specimens from the Germanic Basin because it is heavily crushed and only visible in ventral view, still being embedded in the matrix. Further information on taxonomic assignment is provided in S1 Text, S1 Table. Because the difficulty of assigning humeri of *Nothosaurus* to named species, we first organized the histological and microanatomical description from small to large size, with midshaft width as the size proxy, and second by stratigraphy from oldest to youngest. Possible taxonomic affinities based on histological results are discussed later. In addition to newly sampled specimens, previously sampled humeri are included in the current study [14,23,24].

In addition to the Muschelkalk humeri, a medium-sized humerus of *Ceresiosaurus lanzi* (PIMUZ T4845) from the early Ladinian of Cassina, Meride, Canton Ticino, Switzerland, is included in this study.

### Sampling

The humeri were photographed and measured before sampling (see Table 1). Where possible, bones were sectioned exactly at the narrowest point of the midshaft. However, in some specimens, sampling location is slightly proximal or distal on account of poor bone preservation (Table 1). The thin sections were produced following standard petrographic methods [27]. The entire thin sections were scanned with an Epson V740 PRO high-resolution scanner. In addition, thin sections were studied and photographed with a Leica® DM 750P compound polarizing microscope equipped with a digital camera (Leica® ICC50HD). The bone histological terminology follows Francillon-Vieillot et al. [28]. All thin sections are stored together with the bone they were cut from under the bone's collection number in the respective public collection (see Table 1 and above for list of collections).

Thin sections were drawn by hand from scans, with black denoting vascular spaces and white bone tissue. In these drawings, the ratio of compact bone versus vascular spaces and cavities, i.e., bone compactness (BC in Table 1), was measured with a custom-designed pixel-counting computer program (P. Göddertz, StIPB). Additionally, the drawings were analyzed with the program Bone Profiler [29]. Three parameters provided by this software were used to characterize bone density distribution: C, P, and S (see S2 Table). C is the global bone compactness for the whole sectional area. P is the relative distance from the center of the section to the point where the most abrupt change in compactness is observed. S is the reciprocal of the slope at the inflection point, which generally reflects the width of the transition zone between cortical bone and medullary region. A principal component analysis (PCA) was performed on the parameters cited above in order to explore the distribution of the different taxa in morphospace while optimizing the variance. The analysis was performed on a selection of specimens not affected by distortion/compression, and thus showing a well preserved entire sectional area and inner organization, in order to obtain valid parameter values. Prior to the analysis, all data were Log10-transformed to meet assumptions of normality and homoscedasticity. We tested the influence of size, again using midshaft width (mw) as size proxy, on the various parameters using linear regression analyses. In order to test for differences between specimens based on their stratigraphic origin, on the one hand, and the microanatomical category in which we placed them (see below) on the other, we performed MANOVAs and MANCOVAs (taking size as the co-variable). All of these analyses were performed using the statistic software R (R Development Core Team, 2008). Histological and microanatomical features of the samples are summarized in Table 1.

## Results

### Histological description

#### General histology

The primary cortex of all sampled nothosaur humeri, independent of size or stratigraphic horizon, is basically composed of parallel-fibered bone tissue. The parallel-fibered bone occurs in different degrees of organization and is locally intermixed with woven bone tissue or grades into lamellar bone. Tissue organization varies within a single section as well as between sections but tendentially increases from the inner towards the outer cortex. However, the degree of organization and vascularization can be irregular within a single cross section. The cortex is divided by cyclical growth marks (zones, annuli, and lines of arrested growth), whose analysis will be the topic of another study. Evidence that asymptotic size was reached, or that the individual was most likely close to it, is given by the presence of a distinct layer of lamellar bone visible all around the outer cortex. However, in many samples, a clear reduction in growth rate is only visible locally, whereas other regions of the outer cortex can show continuing growth.

**Fig 1:**
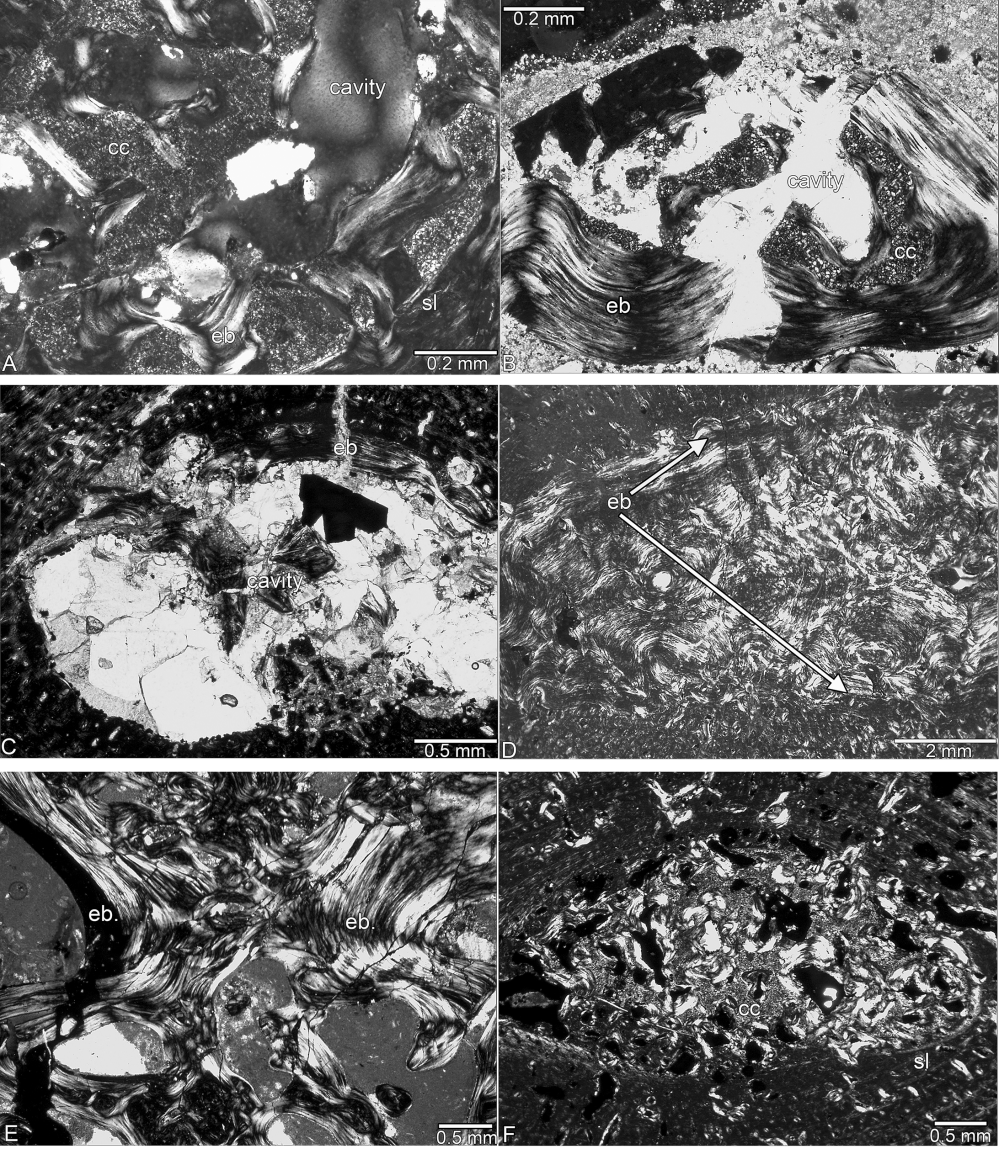
Medullary area in *Nothosaurus* spp. from the Lower and Middle Muschelkalk. (A) Medullary region filled with calcified cartilage and endosteal bone surrounded by a sharp line in SMNS 54317. (B) Medullary region surrounded by a thick layer of endosteal bone and filled by calcified cartilage in Wijk13-259. (C) Medullary cavity incompletely lined by endosteal bone in Wijk13-141. (D) Medullary cavity filled by endosteal bone in MB.R. 780. (E) Medullary region where the two large cavities (Fig 1L) meet in MB.R. 941. (F) Medullary region surrounded by a sharp line and filled by calcified cartilage, endosteal bone yielding erosion cavities in GPIT/RE/1339f, which was sampled distally to midshaft. Abbreviations: cc, calcified cartilage; eb, endosteal bone; sl, sharp line.

**Fig 2:**
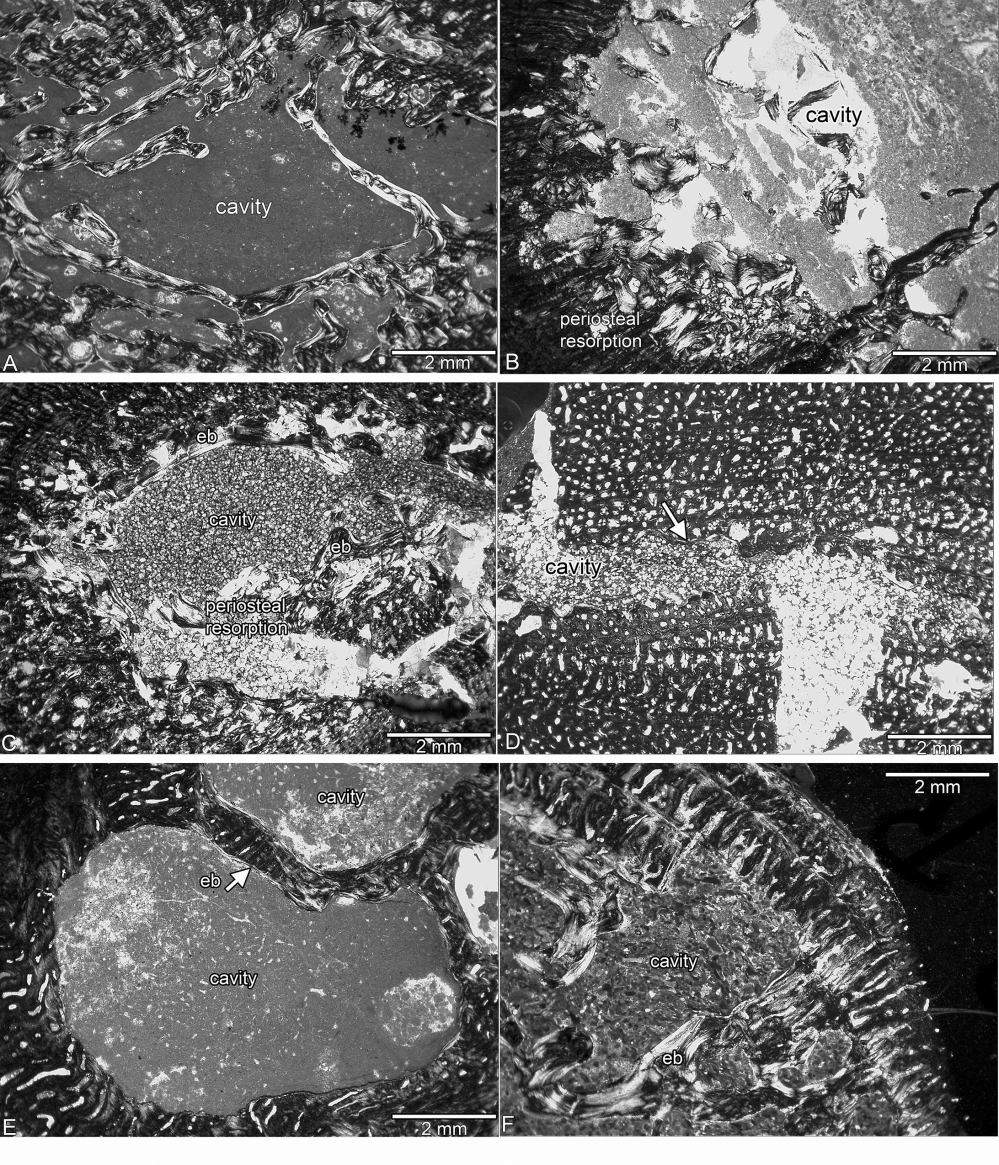
Medullary area in *Nothosaurus* spp. from the Upper Muschelkalk. (A) Free central cavity surrounded by secondary trabeculae indicating ongoing resorption of periosteal bone in SMNS 84772. (B) Large free medullary cavity and resorption of periosteal bone at its margins in SMNS 17214. (C) Free central cavity surrounded by an incomplete layer of endosteal bone and resorption of periosteal bone in MHI 1978. (D) Small free cavity incompletely lined by a thin layer of endosteal bone (arrow) in SMNS 7175. (E) Area of preaxial cavities in MB.R. 270 (Fig 2J). Both cavities are incompletely lined by a thin layer of endosteal bone. (F) Large cavity reaching far into the outer cortex with secondary trabeculae and strong resorption of periosteal bone at its margin in MB.R. 269. Abbreviation: eb, endosteal bone.

**Fig 3:**
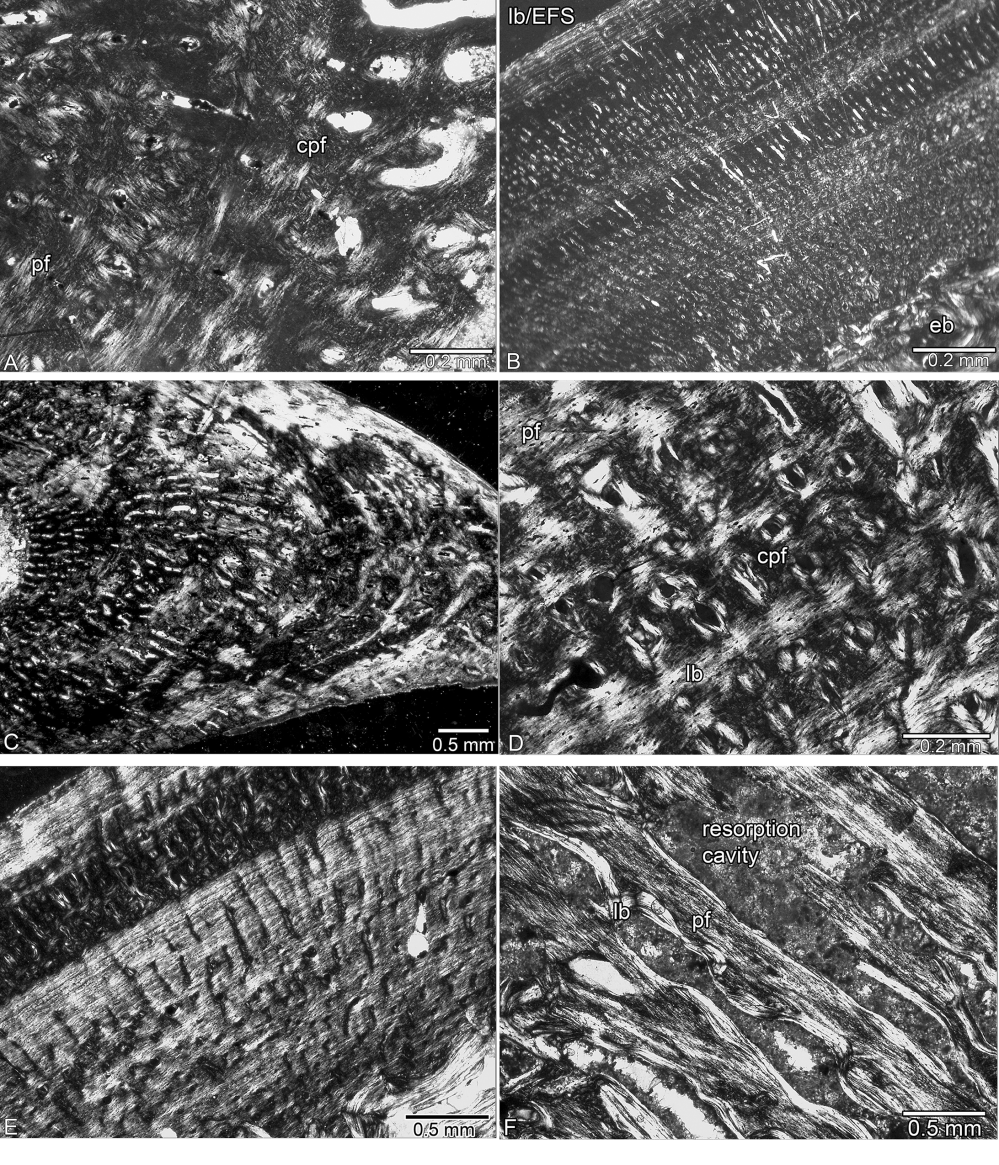
Bone tissue of *Nothosaurus* spp. from the Lower and Middle Muschelkalk. (A) Coarse parallel-fibered and highly organized parallel-fibered bone tissue with simple vascular canals and incompletely lined primary osteons in Wijk 11–87. (B) Alternating zones of fast and slow growth in a compact cortex with a thick layer of lamellar bone deposited in the outer cortex in MB.R. 780. (C) Preaxial half of Wijk13-141 showing alternating layers of parallel-fibered and lamellar bone and predominantly radially arranged vascular canals. (D) Longitudinal primary osteons embedded in coarse parallel-fibered bone, in highly organized parallel-fibered, and lamellar bone tissue in IGWH 7. (E) Alternating layers of coarse and finer, more highly organized parallel-fibered bone tissue in IGWH 25. (F) Resorption of periosteal bone in MB.R. 414. Abbreviations: cpf, coarse parallel-fibered bone tissue; lb, lamellar bone; pf, parallel-fibered bone tissue.

**Fig 4:**
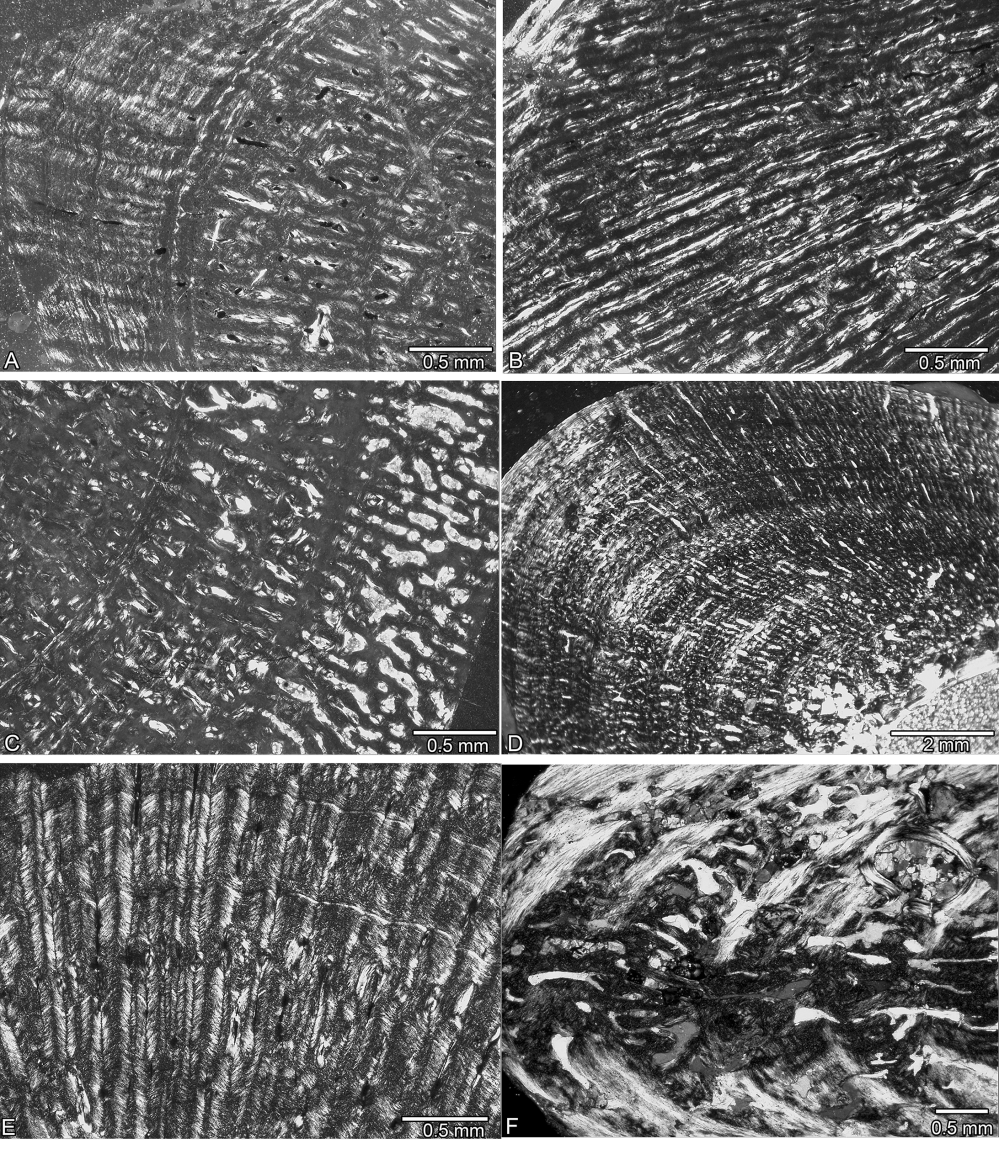
Bone tissue in *Nothosaurus* spp. from the Upper Muschelkalk. (A) Highly organized parallel-fibered bone tissue in the outer cortex and coarse parallel-fibered bone tissue in the inner cortex of GPIT/RE/1590b. (B) Parallel-fibered bone tissue with a strong radial organisation of vascular canals in GPIT/RE/1590d. (C) Parallel-fibered bone tissue with large, irregularly shaped vascular canals in SMNS 2557. (D) Compact bone tissue in MHI 1978. (E) Funnel-shaped arrangement of crystallites around vascular canals in SMNS 17214. (F) Alternating layers of lamellar and coarse parallel-fibered bone tissue and strong resorption of periosteal bone in SMNS 50221.

**Fig 5:**
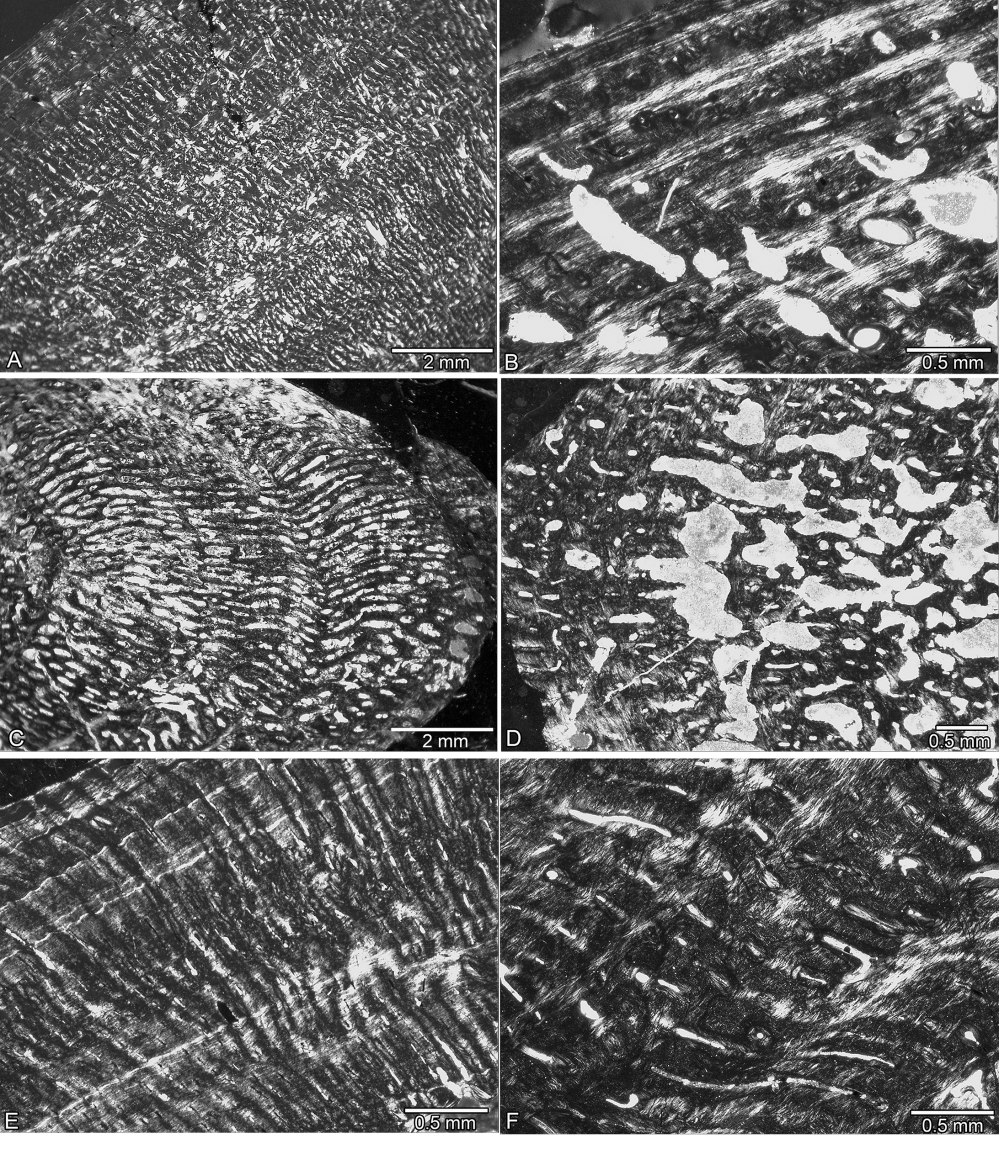
Bone tissue of *Nothosaurus* spp. from the Upper Muschelkalk. (A) Compact bone tissue in SMNS 7175. (B) Resorption of periosteal bone in SMNS 17882. (C) Radial vascular canals in MB.R. 281. (D) Resorption of periosteal bone in MB.R. 278. (E) Alternating layers of coarse and more highly organized parallel-fibered bone tissue in MB.R. 269. (F) Coarse parallel-fibered bone tissue with radial vascular canals in MB.R. 270.

The bone tissue contains small to medium-sized osteocytes. Locally, they may become more numerous and thicker (e.g., in regions with woven or coarse parallel-fibered bone tissue). Longitudinal and radial simple vascular canals dominate the tissue and are arranged in a radial system. In comparison to other Sauropterygia, vascular density is low to moderate [12,16,23–25,30–32]. Many samples show locally or more broadly a funnel-shaped arrangement of crystallites around simple vascular canals [12,24]. The greater the organization of the bone tissue, the more numerous are the funnel-shaped canals. Mature and immature (yet incompletely lined) primary osteons occur in nearly all samples, but their number remains low in Lower and Middle Muschelkalk nothosaurs. Upper Muschelkalk nothosaurs seem to have a higher number of primary osteons. Figs 1 and 2 depict details of the medullary region and Figs 3–5 depict details of the periosteal bone tissue of *Nothosaurus* spp.

#### Lower Muschelkalk samples

SMNS 54317 consists of woven and coarse parallel-fibered bone, as well as more highly organized parallel-fibered bone tissue. Some large radial and longitudinal vascular canals have been transformed into primary osteons. Bone tissue of SMNS 80154 is much more highly organized. The Winterswijk humeri differ among themselves in the degree of tissue and vascular canal organization, and no clear groupings can be discerned. In Wijk13-259, vascular density and the number of osteocytes are low and tissue organization is high. Although larger than Wijk13-259, Wijk11-87 has in its postaxial inner cortex remains of a tissue that contains large simple longitudinal vascular canals together with woven and coarse parallel-fibered bone (Fig 3A). This tissue differs from the rest of the highly organized cortex and is here interpreted as representing an early ontogenetic stage (i.e., possible perinatal tissue). The medullary region of Wijk11-87 contains postaxially and in its middle part calcified cartilage and is surrounded by a sharp line. In Wijk05-9 and TWE 480000320, the entire tissue is made of woven and coarse parallel-fibered bone with large, longitudinal, and mainly simple vascular canals indicating an early ontogenetic stage as well. The more proximal sample of Wijk05-9 shows a sharp line and remains of calcified cartilage at its border, whereas the midshaft sample does not show calcified cartilage or a sharp line. Wijk11-20 differs from the rest due to the dominance of small longitudinal vascular canals. The midshaft and the proximal sample of Wijk11-20 both contain high amounts of calcified cartilage and a sharp line demarcating the medullary region. Wijk13-89, Wijk13-141 (Figs 1C and 3C), Wijk12-91, and Wijk11-20 all show irregular tissue and vascular organization in contrast to the more organized tissue in Wijk10-170 and Wijk11-265. Wijk11-265 shows a high amount of funnel-shaped canals. In Wijk13-141 and Wijk13-89, calcified cartilage and a sharp line demarcating the medullary region are only locally visible. The midshaft samples Wijk12-91, Wijk10-170, and Wijk11-265 do not show any remnant of calcified cartilage or a sharp line; neither do proximal or distal samples of Wijk10-170, and Wijk11-265. Bone tissue organization and vascularity in MB.R. 782 is comparable to the Winterswijk samples. MB.R. 780 has very compact bone tissue with tiny longitudinal vascular canals (Fig 3B). MB.R. 817.1 shows irregularly shaped longitudinal vascular canals. It also shows a large amount of calcified cartilage in the intertrabecular spaces of the medullary region. A clear growth stop indicated by a thick layer of lamellar bone is visible in Wijk13-89, MB.R. 780, and MB.R. 817.1, and maybe growth had nearly stopped in Wijk11-265 and Wijk12-91 (Table 1) (Fig 3B).

#### Middle Muschelkalk samples

The Middle Muschelkalk samples IGWH 25 (Fig 5E) and IGWH 28 are similar in bone tissue organization to the Winterswijk humeri. Except for MB.R. 414 (Fig 3F), the tissue of the remaining Middle Muschelkalk samples is more organized (Fig 3D) than in the Winterswijk specimens (more highly organized parallel-fibered bone, higher amount of lamellar bone), and contains a higher number of funnel-shaped canals. Vascular canals are less radially arranged, more irregular in shape, and are generally larger than in samples from the Lower Muschelkalk. In MB.R. 539 and MB.R. 477, vascularization consists mainly of radial canals. The inner half of the cortex of MB.R. 539 shows parallel-fibered tissue, whereas the outer half largely consists of lamellar bone.

#### In MB.R

414, approximately two-thirds of the cortex is made up of coarse parallel-fibered bone, whereas the outer cortex consists nearly exclusively of lamellar bone that still contains some simple radial vascular canals (Fig 3F). In MB.R. 941, vascular canals are very irregular in size, shape, and arrangement; the specimen contains a high number of primary osteons, which are mainly longitudinally oriented. IGWH 18, IGWH 4, and MB.R. 174.2 show a large amount of calcified cartilage at midshaft. MB.R. 174.2, which was sampled more proximally, shows a sharp line demarcating the medullary region and surrounding a core of calcified cartilage. MB.R. 162.4 shows remains of calcified cartilage in the intertrabecular spaces of the medullary region that surround an open cavity. The medullary region of MHI 1193 shows endosteal bone with the interstitial spaces filled by calcified cartilage. The bone tissue of MB.R. 162.4 and MHI 1193 is more highly organized than in the other samples. A clear growth stop is visible in MB.R. 162.4, IGWH 18, IGWH 4, MB.R. 539, MB.R. 414, and IGWH 17, and growth had nearly ceased in IGWH 25 and IGWH 8 as indicated by an increase in bone tissue organization and decrease of vascular density (Table 1).

#### Upper Muschelkalk samples

The medullary region contains calcified cartilage surrounded by a sharp line in some of the smaller humeri from the Upper Muschelkalk (GPIT/RE/1339d, GPIT/RE/1339f, GPIT/RE/1590c, GPIT/RE/1339c, MHI 1906, and SMNS 2557). A sharp line is missing in GPIT/RE/1590a and GPIT/RE/1339b, but their medullary region also contains large amounts of calcified cartilage (Fig 1F). GPIT/RE/1339a only locally shows remains of calcified cartilage between the intertrabecular spaces. No other nothosaur sample from the Upper Muschelkalk shows calcified cartilage or sharp lines, except for MB.R. 272. In MB.R. 272 remains of calcified cartilage are encompassed within secondary trabeculae. Bone tissue in GPIT/RE/1339c and GPIT/RE/1590c consists largely of coarse parallel-fibered bone, which is interrupted by thin layers of more highly organized tissue. In GPIT/RE/1590c, the primary cortex forms only a thin layer around a large core of calcified cartilage.

Except for GPIT/RE/1339a and GPIT/RE/1590d, the histology of the GPIT/RE/ sample is similar to that of the humeri from Winterswijk. GPIT/RE/1590d and MHI 633 are dominated by a radial vascular organization (Figs 4B and 5C). Radial vascular canals also occur in SMNS 81884, SMNS 53012, and SMNS 2557, but in these samples it is restricted to the preaxial half. MHI 1906 differs from the others because it postaxially has a high number of large open, round to oval vascular canals that are mainly arranged radially. SMNS 50221 has a low primary vascular density, and MB.R. 282 and SMNS 84772 have only a moderate one. The spongy appearance of these bones is due to secondary resorption of the periosteal cortex (Figs 2A, 2F, 4F, 5B and 5C).

The bone tissue of SMNS 50221 (Fig 4F), SMNS 17214 (Fig 4E), MB.R. 282, MHI 1978 (Fig 4D), MB.R. 279, MB.R. 272, StIPB R54/2, StIPB R 53, StIPB R 45, and SMNS 84772 is more organized than the previously discussed Upper Muschelkalk samples. It contains a higher amount of highly organized parallel-fibered and lamellar bone and more funnel-shaped vascular canals. Overall vascular density is moderate in these samples. MHI 754, SMNS 84851, MB.R. 278, MB.R. 281, SMNS 80688, SMNS 81988, SMNS 17882, MB.R. 270, PIMUZ AIII-1, PIMUZ AIII-2, StIPB R 40, and MHI 873 show coarse parallel-fibered and poorly organized parallel-fibered bone tissue with numerous large vascular canals (often already partially resorbed), regularly interrupted by layers of more organized tissue. SMNS 7175 has very regular bone tissue with a large number of small, mainly longitudinal vascular canals, of which many are already filled by lamellar bone (Fig 5A). MB.R. 269 shows a high amount of lamellar bone throughout its entire cortex but has locally areas with poorly organized parallel-fibered bone tissue and a high primary vascular density (although this was already locally secondarily altered) (Fig 5E). A clear growth stop is visible in GPIT/RE/1339f, GPIT/RE/1590b, SMNS 50221, SMNS 84772, MB.R. 282, StIPB R 53, and SMNS 17882. Specimens GPIT/RE/1590a, SMNS 17214, SMNS 84851, MHI 754, MHI 1978, SMNS 80688, SMNS 7175, MHI 873, MB.R. 270, MB.R. 269, StIPB R 45, StIPB R 54/2, PIMUZ4845, PIMUZ AIII-1, PIMUZ AIII-2, and MB.R. 272 were presumably nearly fully grown as well (Table 1).

#### Ceresiosaurus lanzi

The histology of the large humerus of *Ceresiosaurus lanzi* (PIMUZ T4845) consists of parallel-fibered and lamellar bone, which contains radial and longitudinal vascular canals. The nature of this bone tissue cannot be determined due to the excessive thickness of the thin section. Locally, large open radial vascular canals are numerous, although most of the cortex is not well vascularized and compact.

### Resorption and remodeling

#### Lower Muschelkalk samples

In SMNS 80154, a thick layer of endosteal bone borders most of the medullary cavity. The medullary cavity of most Winterswijk samples is only incompletely lined by endosteal bone. In Wijk13-259 (ventrally) and Wijk12-91 (ventrally), endosteal bone forms locally thick but still incomplete layers (Fig 1B). In Wijk13-89 and Wijk11-20, the medullary cavity is completely lined by a layer of endosteal bone. Active resorption seems to have stopped in the completely filled medulla of MB.R. 780. SMNS 54317 (pre- and postaxially), Wijk10-170 (preaxially), Wijk12-91 (preaxially and ventrally), Wijk11-20 (mainly ventrally), and MB.R. 817.1 (postaxially) show some resorption of periosteal bone in the inner cortex. No resorption of periosteal bone is visible anywhere in the cortices of MB.R. 782, Wijk13-259, Wijk05-9, Wijk13-141 (Fig 3C), and Wijk11-265.

#### Middle Muschelkalk samples

The medullary cavities of IGWH 3, IGWH 25, MB.R. 162.4, MB.R. 477, MHI 1193, and MB.R. 539 are completely lined by endosteal bone, whereas those of IGWH 11, IGWH 12, IGWH 14, IGWH 18, IGWH 8, IGWH 17, MB.R 414, IGWH 4, and MB.R 941 are only incompletely lined (Fig 1E; Table 1). Finally, the medullary cavities of MB.R. 174.2, IGWH 28, and IGWH 7 lack a lining by endosteal bone. No resorption of periosteal bone occurs in the cortices of IGWH 3, IGWH 28, IGWH 14, IGWH 7, IGWH 18, IGWH 8, and MB.R. 539. Scattered erosion cavities in the inner cortex are present in IGWH 11, IGWH 12, IGWH 25 (mainly preaxially), MB.R. 174.2 (all around), IGWH 17 (pre- and postaxially), IGWH 4 (postaxially), MB.R. 162.4 (pre- and postaxially), MB.R. 477 (pre- and postaxially), and MB.R. 941 (pre- and postaxially). MB.R. 414 differs from the other samples in the strong resorption of periosteal bone (Fig 3F), mainly in the preaxial half of the humeral cross section. In this sample, simple vascular canals and primary osteons were enlarged by resorption, resulting in large cavities. Many of these resorption cavities are lined by thick layers of secondary lamellar bone, but considerable re-deposition of bone (resulting in a complete infilling of the cavities) is not visible.

#### Upper Muschelkalk samples

None of the Upper Muschelkalk specimens displays a complete lining of the medullary cavity by endosteal bone. The medullary cavities are incompletely lined by endosteal bone in GPIT/RE/1590d, SMNS 53012, MHI 754, SMNS 84851, and MHI 1978. The inner cortex of MHI 1906 and GPIT/RE/1339b shows balanced resorption and redeposition of endosteal bone. In GPIT/RE/1590a, GPIT/RE/1590b (preaxially), GPIT/RE/1339d, and GPIT/RE/1339f, redeposition of bone was higher than resorption, resulting in a very compact medullary region. In GPIT/RE/1339a, resorption in the inner cortex is higher than redeposition, resulting in a large, not clearly delimited medullary region.

Resorption of periosteal bone does not occur in GPIT/RE/1339c, MHI 633, MHI 1906, GPIT/RE/1590d, GPIT/RE/1339b, GPIT/RE/1339d, GPIT/RE/1590c, SMNS 53012, SMNS 2557, GPIT/RE/1339f, SMNS 84851 (nearly none), SMNS 81884, and SMNS 7175 (nearly none). Resorption is also limited in MB.R. 269, SMNS 17214, SMNS 81988, MHI 873, StIPB R 40, StIPB R 53, StIPB R 54, PIMUZ AIII-1, and MB.R. 279. Resorption of the inner cortex in form of scattered erosion cavities close to the medullary region is visible in GPIT/RE/1590a, GPIT/RE/1590b, SMNS 80688, MHI 754 (ventrally and preaxially), and MHI 1978 (preaxially). In all other samples, resorption of periosteal bone reaches far into the outer cortex (Fig 5B and 5D). Here, resorption of periosteal bone starts by an enlargement of mainly radial vascular canals (MB.R. 281 [preaxially] and MB.R. 278 [postaxially]) and results in large cavities scattered throughout the entire cortex (PIMUZ AIII-2, SMNS 17882, MB.R. 272). SMNS 50221, SMNS 84772, and MB.R. 282 are rather special because they show extremely strong resorption of periosteal bone all over the cortex without much redeposition of secondary bone. This results in a spongy periosteal tissue, similar to the Middle Muschelkalk sample MB.R. 414.

Ceresiosaurus lanzi—The large humerus of *Ceresiosaurus lanzi* (PIMUZ T4845) shows no resorption of periosteal bone.

### Microanatomical description

#### Lower Muschelkalk samples

**Fig 6:**
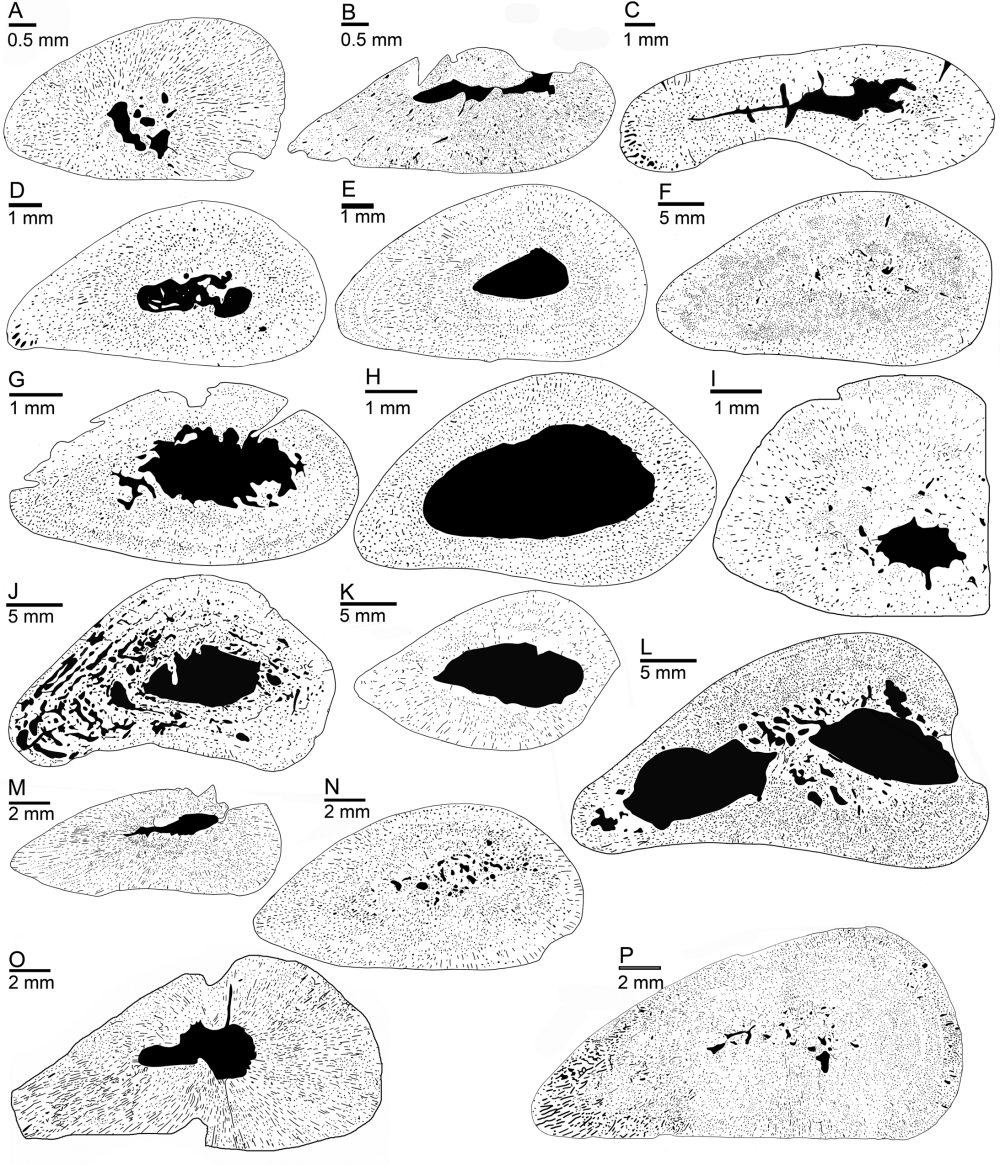
Humeral microanatomy of *Nothosaurus* spp. (A-F) Black and white drawings of cross sections of Lower Muschelkalk samples. (A) SMNS 54317. (B) Wijk13-259. (C) Wijk11-87. (D) Wijk13-141. (E) Wijk11-265. (F) MB.R. 780. (G-L) Black and white drawings of cross sections of Middle Muschelkalk samples. (G) IGWH 25. (H) IGWH 7. (I) MB.R. 162.4. (J) MB.R. 414. (K) MB.R. 539. (L) MB.R. 941. (M-P) Black and white drawings of cross sections of Upper Muschelkalk samples. (M) GPIT/RE/1590d. (N) GPIT/RE/1339f. (O) MHI 633. (P) SMNS 2557.

SMNS 54317 has a spongy medullary region that is filled by calcified cartilage and endosteal bone (Fig 1A). The medullary cavity in SMNS 80154 is very small and additionally filled by endosteal deposits. The humeri from the locality of Winterswijk all share a free medullary cavity at midshaft surrounded by a compact cortex (Figs 1C, 6A–6E; Table 1). However, despite a similar general internal structure, the relative size of these cavities and of the medullary area more generally is variable. MB.R. 782 has a small free cavity that is surrounded by a thick layer of endosteal bone, which also coats the inner parts of the nutrient canal. MB.R. 780 is peculiar in having a medullary region completely filled by endosteal bone, resulting in an extremely compact section (Figs 1D and 6F). MB.R. 817.1 shows a spongy and rather large medullary region (in accordance with its more distal sampling location by comparison with other specimens in this study) with large and randomly shaped cavities and secondary trabeculae. Most of the Lower Muschelkalk samples show relatively high bone compactness, with values above 80%; only Wijk05-9 (from a probably juvenile specimen) and the distally sectioned MB.R. 817.1 display compactness values below 80% (Table 1).

#### Middle Muschelkalk samples

Some samples from the Middle Muschelkalk share a large medullary cavity, including IGWH 3, MB.R. 162.4, MB.R. 174.2, IGWH 14, and IGWH 7. MHI 1193, MB.R. 162.4, and MB.R. 477 differ from the other Middle Muschelkalk samples in their small medullary cavity which is off-center in the latter two (Fig 6H–6L; Table 1). Other samples (IGWH 11, IGWH 12, IGWH 17, MB.R. 941) show two or more large and several randomly sized and shaped cavities separated by endosteal bone (Table 1). IGWH 18 and IGWH 4 both show a medullary region filled by calcified cartilage, which encompasses large cavities. Except for MB.R. 174.2, MB.R. 162.4, MB.R. 477, and IGWH 3, all samples from the Middle Muschelkalk have bone compactness (BC) values below 80% (Table 1). IGWH 14 has the lowest value (43%) among the Middle Muschelkalk sample; this is comparable to the low BC values of some large-bodied nothosaurs from the Upper Muschelkalk (Table 1).

#### Upper Muschelkalk samples

**Fig 7:**
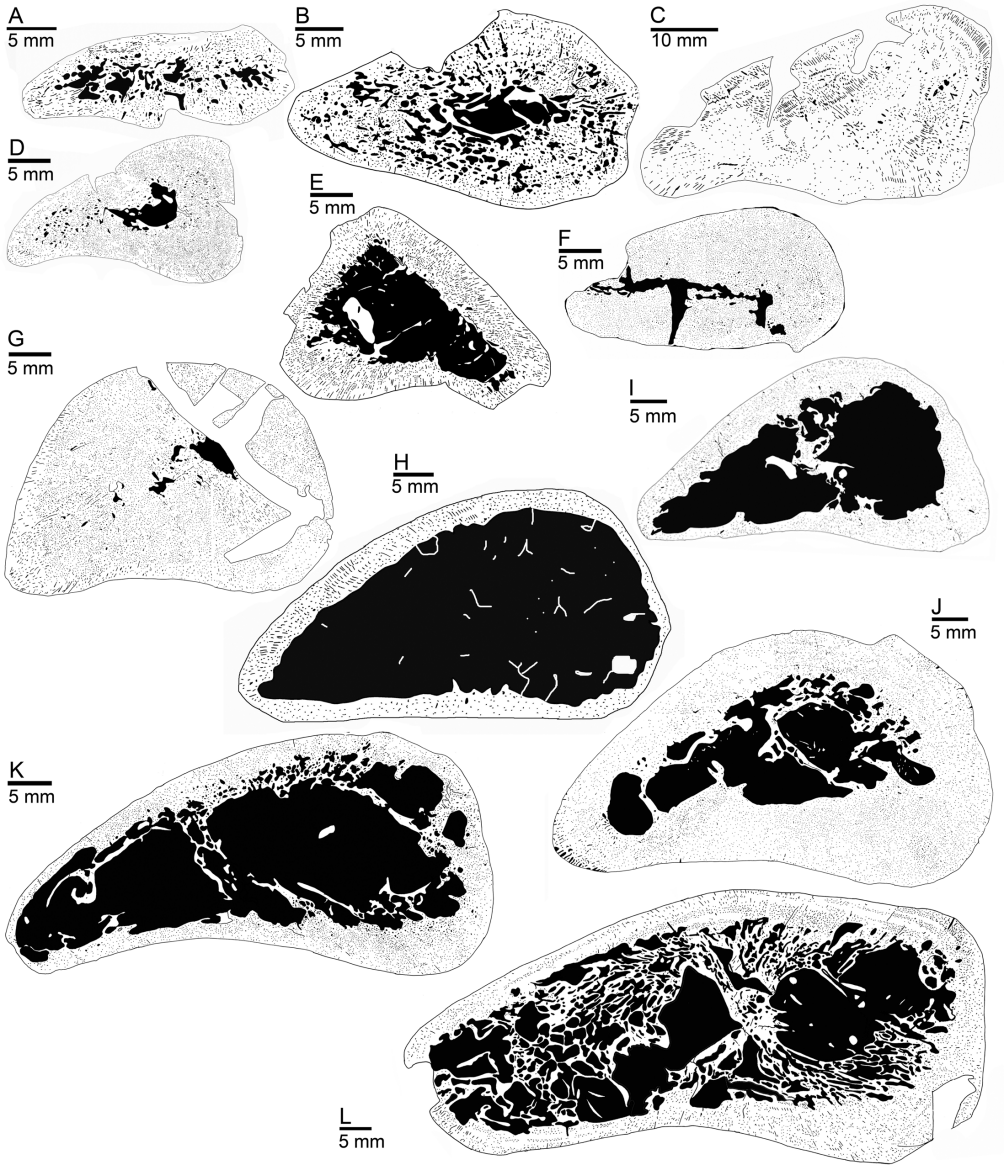
Humeral microanatomy of *Nothosaurus* spp. and of *Ceresiosaurus lanzi*. Black and white drawing of cross sections. (A) SMNS 50221. (B) SMNS 84772. (C) PIMUZ T 4845 (*Ceresiosaurus lanzi*). (D) MHI 1978. (E) SMNS 17214. (F) SMNS 84851. (G) SMNS 7175. (H) PIMUZ A001. (I) MB.R. 279. (J) MB.R. 270. (K) MHI 873. (L) MB.R. 269.

The Upper Muschelkalk nothosaur sample spans the entire range of very compact (BC of 93.3% in GPIT/RE/1590b) to spongy and thin-walled bones (BC of 23.3% in StIPB R 53) (Table 1). Some sections are highly compact and show no free medullary cavity (GPIT/RE/1339d, GPIT/RE/1339f, SMNS 2557, SMNS 7175), or only a relatively small free medullary cavity (GPIT/RE/1590b; PIMUZ T4845, GPIT/RE/1590d) (Figs 6M–6P and 7). In MHI 633 and MHI 1978, the medullary cavity is of moderate size. The medullary area consists of several cavities in GPIT/RE/1590a, MHI 1906, and GPIT/RE/1339b. In GPIT/RE/1590b and SMNS 7175, the process of filling the medullary cavity by endosteal bone is at an early stage, whereas it is nearly completed in GPIT/RE/1339d, GPIT/RE/1339f, and SMNS 2557.

Large (SMNS 81884, SMNS 17214, SMNS 17882, MHI 754, MB.R. 281) and very large (SMNS 81885, MB.R. 269, SMNS 81988, MHI 873, StIPB R 40, StIPG R 45, StIPB R 53, StIPB R 54/2, PIMUZ AIII-1, PIMUZ AIII-2, MB.R. 279, MB.R. 278, MB.R. 272) medullary cavities occur only in samples from large-bodied nothosaurs (midshaft width >40 mm; Table 1) (Fig 7). The large cavities often contain local remains of primary periosteal bone, endosteal bone, and pieces of broken secondary trabeculae, indicating damage of the inner structure. Only in MB.R. 269, the entire medullary cavity is traversed by a system of secondary trabeculae (Fig 7L). MHI 873, MB.R. 272, PIMUZ AIII001, PIMUZ AIII002, StIPB R 40, SMNS 81885, and StIPB R 53 have the thinnest cortices among the sample (BC <40%). Some samples show a moderately-sized medullary region (occupying the inner third to inner half of the section: GPIT/RE/1590a, GPIT/RE/1339b, MHI 1906) or enlarged medullary region (occupying over half of the section: GPIT/RE/1339a, SMNS 50221).

#### Ceresiosaurus lanzi

The humerus of *Ceresiosaurus lanzi* (PIMUZ T4845) has very compact cortical tissue, and the medullary region is nearly completely filled by endosteal deposits (Fig 7C).

#### Results of principal component analysis (PCA)

**Fig 8:**
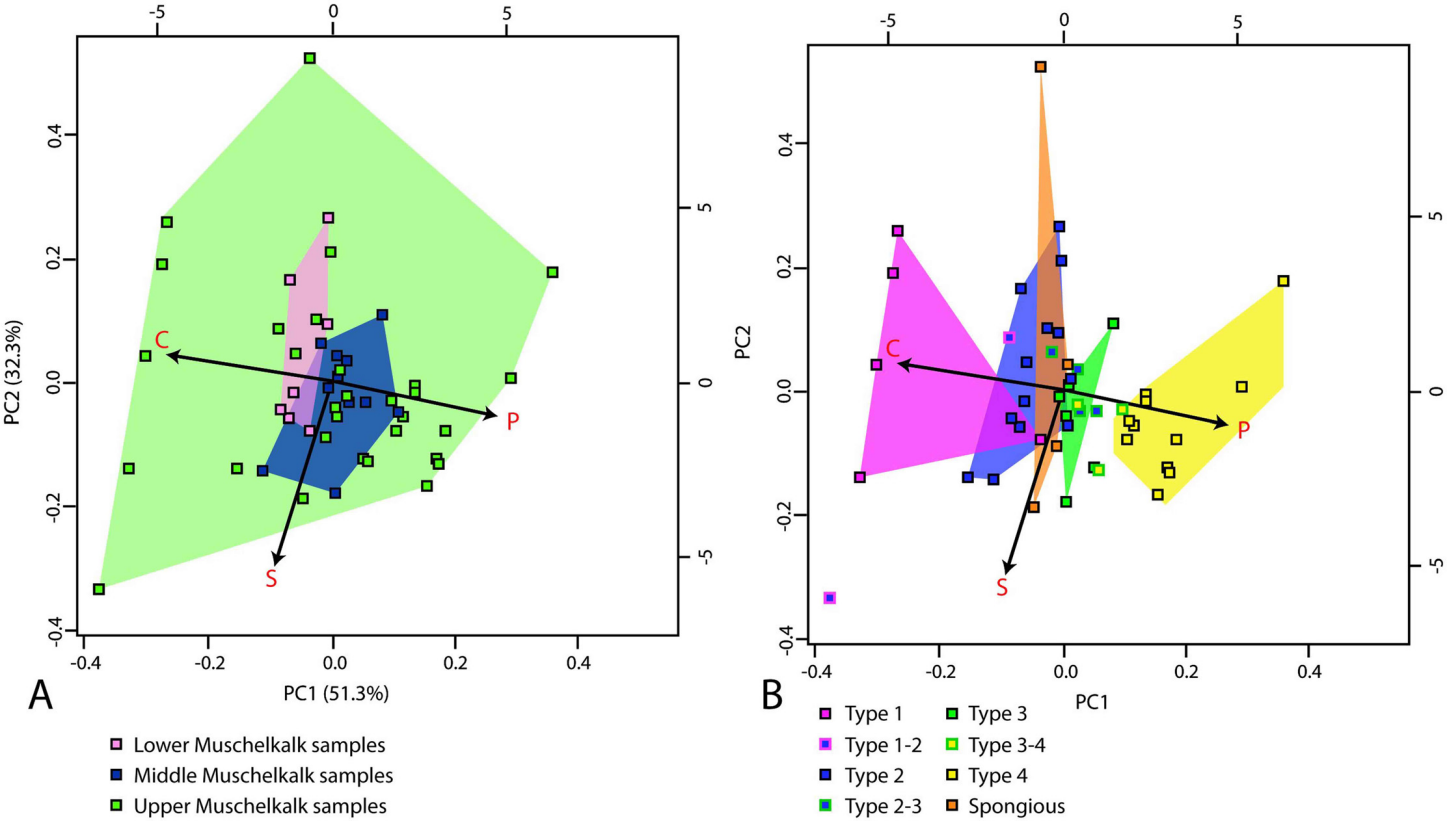
Microanatomical clusters obtained by PCA conducted on a sample of the humerus cross section of *Nothosaurus* spp. (A) Graph showing the distribution of the variance according to the stratigraphic origin of the specimens. (B) Graph showing the distribution of the variance according to the microanatomical category of the specimens (see discussion).

The PCA shows that the two main axes explain most of the variation (83.6%; Fig 8). While the Upper Muschelkalk specimens cover the whole variation observable in the *Nothosaurus* spp. sample (Fig 8A), the Lower and Middle Muschelkalk samples occupy more restricted distribution areas. Interestingly, they are rather distinct from each other despite some limited overlap. C (compactness) and P (point of most abrupt change in compactness) are the main variables discriminating the taxa along the first PCA axis (Fig 8A). Conversely, the width of the transition zone (S) discriminates specimens along the second axis. Although these trends are naturally associated with general bone microanatomical features (Fig 8B), no clear grouping is observed. This reflects the continuous variation in *Nothosaurus* microanatomical features and the gradual transitions between the major microanatomical types (Fig 8B). It is worth noting that S has no real impact on the distinction between the various microanatomical types in our *Nothosaurus* sample.

Bone Profiler is a software that was designed to analyze microanatomy in bone sections that have a vaguely circular outline and two concentric zones. The inner structure of the nothosaur sample is very variable and many of the large samples show a rather random distribution of large resorption cavities with no distinguishable medullary area (e.g., MB.R. 414, MB.R. 282, SMNS 50221, SMNS 84772, and GPIT/RE/1339a), resulting in the values attributed to the S and P parameters having no structural meaning. The specimens showing a thin-walled cross section are nevertheless clearly distinct from the ones having a thick and compact cortex (Fig 8B).

#### Tests on the microanatomical parameters

Linear regressions show the influence of size (medullary width) on C (r = -0.59, p <0.001) but not on S (r = 0.07, p = 0.65) and P (r = 0.16, p = 0.29). The MANOVAs and MANCOVAs similarly show significant differences in the values of the microanatomical parameters C, S, and P based on both stratigraphic origin (Lower, Middle or Upper Muschelkalk; p <0.01) and microanatomical category (p <0.01).

## Discussion

### Choice of sampling location

In bone histological and microanatomical studies, it is crucial to compare homologous sections because of the variation in microanatomy throughout the bone caused by developmental origin of the tissue and morphogenesis. Thus, thin sections of long bones were usually taken where the midshaft is the narrowest, because this represents the plane intercepting the growth center (center of ossification) or neutral zone [12,25,32–35]. All our sections are thus comparable among nothosaurs and with those of other taxa, notably among other Sauropterygia [12,16,23–25,30–32,36]. In nothosaurs, the nutrient canal as the indicator for the position of the growth center [37,38] is not reliable. The nutrient foramen, i.e., the opening of the canal to the outer bone surface, is located at the transition from the proximal head to the midshaft, below the deltopectoral crest. All samples of *Nothosaurus* were taken distal to the nutrient foramen, and only some thin sections show a canal in the cortex that might be identifiable as part of the nutrient canal and which is intersected in different planes (e.g., SMNS 54317, MB.R.782, GPIT/RE/1339a, MHI 754, GPIT/RE/1339b, GPIT/RE/1339d). The growth center, however, is at midshaft in nothosaurs as indicated by no or limited remodeling of periosteal bone at the narrowest midshaft, whereas samples that were taken more proximally (closer to the nutrient canal) or further distally display distinctly more endosteal remodelling. The absence of calcified cartilage and the absence of the sharp line representing the boundary between the endosteal and the periosteal domains further indicate the narrowest midshaft as the area that has the most complete growth record in *Nothosaurus*. This is also supported by a large, serially sectioned humerus of *Nothosaurus* from the Upper Muschelkalk from its proximal head to its distal end [24]. The thin sections from the region of the nutrient canal [24] show less primary cortex preserved than the midshaft thin section [24]. Although microanatomy varies along the shaft, this series of thin sections furthermore documented that the structure remains essentially constant (tubular with a very large but not well delimited medullary region). This is contrary to what is observed in the stratigraphically older *Nothosaurus* specimens, where a free cavity at midshaft proximally and distally turns into spongy bone.

Independent of which region of the midshaft is compared; variation in sampling location along midshaft alone cannot explain the high microanatomical variability observed in the current sample of *Nothosaurus*, specifically in the Upper Muschelkalk forms that show a wide range of relative cortical thicknesses.

### Calcified cartilage

In *Nothosaurus* spp., the presence of calcified cartilage depends on sampling location along the shaft as well as on ontogeny, and on intra- and interspecific differences in endochondral ossification. In adult Lower and Middle Muschelkalk nothosaurs, calcified cartilage persists in some individuals at the narrowest part of the midshaft, but not in others (Table 1). When present, remains of calcified cartilage are embedded in the endosteal bone. None of the midshaft samples keep an entire core of calcified cartilage, as opposed to the more distal samples (Table 1). Small humerus SMNS 54317 and some of the smaller humeri (mw <25 mm) from the Upper Muschelkalk show a core of calcified cartilage at midshaft, indicating an early ontogenetic stage. Among the larger humeri (mw >25 mm) from the Upper Muschelkalk, only one (MB.R. 272) shows locally remains of calcified cartilage.

Calcified cartilage at midshaft resulting from incomplete endochondral ossification is a typical feature of osteosclerosis [39], as seen in adults of the stratigraphically somewhat younger pachypleurosaurs from the Alpine Triassic [31]. However, few of the adult sampled *Nothosaurus* specimens show this histological feature, which does not seem to have been fixed during nothosaur evolution.

### General histological observations and resorption activity

Samples of *Nothosaurus* spp. may show woven bone in combination with some primary osteons or incompletely lined primary osteons. This tissue can then be interpreted as incipient fibrolamellar bone or true fibrolamellar bone, respectively [12,23,24]. However, fibrolamellar bone tissue of *Nothosaurus* spp. is always restricted to layers or regions and never forms the entire cortex, as opposed to some other Triassic Sauropterygia [24,25,32,40,41]. Coarse parallel-fibered bone tissue is more common than woven bone. This bone tissue was already described for several other aquatic tetrapods (the temnospondyl *Plagiosaurus*, [42]; mosasaurs, [43]; ichthyosaurs, [44]; placodonts, [25,32]; *Simosaurus*, [16]). Organization of this tissue together with a high density of osteocytes indicates fast deposition and thus suggests increased growth rates when compared to the deposition of typical parallel-fibered bone tissue [16].

*Nothosaurus* spp. share with other sauropterygian taxa such as placodonts [25,32], pachypleurosaurs [12,30,31,36], pistosaurs [24], and plesiosaurs [24,41] dominantly radially organized vascularization as expressed by a high amount of radial vascular canals in addition to longitudinal ones. The exception is the nothosaur *Simosaurus*, which does not show radial vascular organization [16]. Independently of stratigraphic age or size, many *Nothosaurus* samples show preaxially a high number of large radial vascular canals, indicating faster growth in this region than elsewhere. Radial vascularity similar to that of sauroptergyians was also described for early synapsids [45,46], indicating that this tissue is not restricted to marine reptiles.

Cortical resorption patterns are highly variable in *Nothosaurus*, ranging from nearly no resorption to strong resorption, the latter resulting in BMD. The resorption activity in some large-bodied *Nothosaurus* spp., resulting in very thin-walled humeral cross sections, is unique in its intensity and absence of secondary deposits among other marine reptiles or any other amniote except birds, as already pointed out by Krahl et al. [24].

In a few samples (SMNS 84772, SMNS 50221, MB.R. 282, MB.R. 414), much of the primary cortex is resorbed in a peculiar pattern, resulting in large irregular cavities and in a spongy periosteal tissue, whereas the medullary cavity—if distinguishable at all—retains a moderate size. Resorption in these specimens did not follow the standard pattern starting from the inner cortex and continuing into the outer cortex, but occurred randomly throughout the cortex.

Because of the lack of any modern analogue for these two different but unusual resorption activities, their meaning remains unresolved. Biomechanical explanations and those invoking reproductive or physiological stresses (e.g., salinity fluctuations) must remain hypothetical.

### Microanatomical patterns in *Nothosaurus* spp.: Increase vs. decrease in bone mass

Bone mass increase (BMI) [47] is a mechanism by which secondarily aquatic tetrapods living in shallow waters increase their specific bone density to counteract the positive buoyancy caused by the lungs [39]. BMI is achieved through osteosclerosis, which can be the result of different mechanisms, such as incomplete endochondral ossification (i.e., persistence of calcified cartilage), the filling of inner cavities by excessive secondary bone deposits, and/or the inhibition of cortical resorption [39,47]. Osteosclerosis, often in combination with morphologically observable pachyostosis, has been described for many marine reptiles and some marine mammals that are interpreted as slow swimmers moving about by long but shallow dives [39,48–50]. In *Nothosaurus*, pachyostosis has been documented only in ribs [1], which have not been histologically studied yet. *Ceresiosaurus* humeri show pachyostosis and osteosclerosis [23] (this study). Bone mass decrease (BMD) is caused by an imbalance between strong resorption and limited secondary deposits. BMD occurs in pelagic forms, such as some marine turtles, mesosuchian marine crocodilians, ichthyosaurs, adult plesiosaurs, mosasaurs, and modern cetaceans [39,47]. It seems to be typical for active swimmers requiring high speed and maneuverability in open water habitats. However, although these groups also show BMD, it is reached in *Nothosaurus* in a higher degree and maybe by different processes involved.

To evaluate if a taxon shows BMI or BMD, the condition seen in its ancestor must be considered. However, the terrestrial ancestor of marine Sauropterygia is not known [1], and their relationships within higher diapsids are largely unresolved [25]. Nevertheless, terrestrial amniotes usually have a large free medullary cavity surrounded by a layer of compact cortex [51–53]. Long bones of *Claudiosaurus*, a Late Permian neodiapsid and a putative morphological precursor of Triassic eosauropterygians, has a small medullary cavity surrounded by a thick cortex, displaying osteosclerosis [35,39]. When compared to similar sized *Nothosaurus* spp., *Simosaurus* [16] and *Ceresiosaurus* [23] both show smaller medullary cavities surrounded by a relatively thicker, compact cortex and thus also display osteosclerosis. Microanatomy of European pachypleurosaurs is well known [30,31,35] and varies within a single taxon as well as between taxa: The pachypleurosaur *A*. *heterodontus* from the early to middle Anisian shows a comparable variable size range of the free medullary cavity when compared to *Nothosaurus* from the same locality but a generally higher vascular density, resulting in lower bone compactness [30] (Table 1). Pachypleurosaurs from the Ladinian of the Alpine Triassic (*Neusticosaurus* spp.; *Serpianosaurus*) have a small medullary cavity or show incomplete endochondral ossification [31,35,39]. These pachypleurosaurs document stronger osteosclerosis, when compared to *A*. *heterodontus* and *Nothosaurus* as do Placodontia [25,39] and Pistosauria [24]. Thus, Triassic Sauropterygia, except for some medium-sized to large-bodied individuals of *Nothosaurus*, principally display osteosclerosis and BMI. Modern semi-aquatic crocodiles share with large-bodied *Nothosaurus* a similar dorsoventrally flat body shape and size range. Based on illustrations of thin sections of humeri and femora [54,55] crocodiles show, contrary to most large-bodied *Nothosaurus*, osteosclerosis and therefore BMI. A distinct decrease in bone mass as observed in nothosaurs with thin-walled cortices may play an important role in adding positive buoyancy for surface swimmers. Surface swimming was also suggested for some small, marine squamates (dolichosaurids) that show much lightened vertebrae and ribs [56].

The microanatomical patterns seen in *Nothosaurus* spp. are subdivided in Table 1 into four categories. Category 1 refers to true BMI, with samples showing a small medullary cavity (i.e., reduced when compared to a hypothetical terrestrial ancestor) surrounded by a thick cortex of compact bone. Samples representing category 2 have a moderately sized medullary cavity surrounded by compact bone. Category 3 refers to samples with an enlarged medullary cavity surrounded by compact bone. This category is comparable to the microanatomy of humeri of modern varanids [57,58] and iguanines [59] but when compared to other Triassic Sauropterygia [1] this category documents a decrease in bone mass. Category 4 represents true BMD, because samples have an extremely thin-walled humeral cross section. These microanatomical categories were statistically tested and found significant.

#### Lower Muschelkalk samples

Compared to modern sauropsids and to the entire *Nothosaurus* sample, samples from the Lower Muschelkalk of Winterswijk generally show BMI (i.e., osteosclerosis; category 2, Table 1) and are comparable to Pachypleurosauria, Placodontia, *Simosaurus*, and *Ceresiosaurus*, although their medullary cavities are tendentially larger. An exception is the large humerus MB.R. 780 from the Lower Muschelkalk of Poland that shows an extreme reduction of the medullary cavity because it is completely filled by endosteal bone (BMI or category 1; Table 1). Bone compactness is here comparable to small pachypleurosaurs from the Alpine Triassic [31]. The small humerus MB.R. 782 from the Lower Muschelkalk of Poland has a reduced medullary cavity, and filling of the remaining cavity by endosteal bone was in progress when the animal died. Contrary to these samples, MB.R. 817.1 also from the Lower Muschelkalk of Poland shows high endosteal resorption rates, resulting in a spongy medullary region surrounded by a locally thick and compact cortex (category 2; Table 1). The various osteosclerotic microanatomies of these Lower Muschelkalk nothosaurs thus fit environmental conditions that would have been near-shore to shallow marine (S1 Text, S1 Table).

#### Middle Muschelkalk samples

In the Middle Muschelkalk sample, two seemingly opposing patterns are documented. MB.R. 174.2, MB.R. 477, and MB.R 162.4 show BMI achieved by presence of calcified cartilage and moderate endosteal resorption with deposition of secondary bone (category 2; Table 1). The rest of the Middle Muschelkalk sample shows medium-sized to large free medullary cavities (category 3; Table 1) when compared to the nothosaurs from the Lower Muschelkalk. Comparing them to modern sauropsids, one could argue that these specimens are possibly closest to the condition of the unknown terrestrial ancestor. However, these specimens more likely represent a trend towards BMD, away from the osteosclerotic condition of the Lower Muschelkalk nothosaurs and that of placodonts, pachypleurosaurs, and pistosaurs. A third microanatomical category is represented by MB.R. 414 with its spongy periosteal tissue, possibly representing a different pathway towards BMD.

The localities from which all these Middle Muschelkalk samples originate were close to the center of the basin with seemingly deeper water, but the Middle Muschelkalk is characterized by sea level regression (S1 Text). The presence of islands, fluctuating sea level, and taphonomic processes such as redeposition and accumulation of bones from different habitats as well as migration of marine reptiles into the Germanic Basin from more pelagic environments (i.e., from the Tethys through the temporarily open gates) as well as possible developmental plasticity or a sexual dimorphism might explain this strong variation in microanatomy.

#### Upper Muschelkalk samples

Several samples from the Upper Muschelkalk document BMI in different degrees of intensity (category 1, 2; Table 1) when compared to the *Nothosaurus* sample in general as well as to other Sauropterygia. This was achieved by different processes: remodelling inhibition, incomplete ossification/presence of calcified cartilage at midshaft, filling of the medullary region by endosteal bone, and excessive secondary bone deposits. In addition to the samples exhibiting BMI, many large humeri show BMD (category 3, 4; Table 1). As already noted, there are two ways of achieving BMD in the larger *Nothosaurus* humeri from the Upper Muschelkalk: a general reduction in compactness by resorption vs. an increase in the size of the free medullary cavity. As described in S1 Text, the Upper Muschelkalk was a phase of transgression with a sea level high stand. The increase in microanatomical diversity is accompanied by an increase in taxonomical diversity [1,9]. However, developmental plasticity and sexual dimorphism might be considered as well.

### Taxonomic implications

A taxonomic classification of nothosaur humeri based on morphology remains inconclusive as discussed above, raising the question whether histology or microanatomy allow a more precise taxonomic assignment.

Two *Nothosaurus* species are known from the Lower Muschelkalk of Winterswijk: the diminutive *N*. *winkelhorsti*, and the small-bodied *N*. *marchicus* [14,60,61]. Recently, the small nothosaurid *Lariosaurus vosseveldensis* was also described from the locality of Winterswijk [62]. Despite their small size, humeri Wijk13-259 and SMNS 80154 do not represent early ontogenetic stages based on bone tissue organization and vascularization pattern. They thus seem to represent a smaller-bodied taxon than *N*. *marchicus*; likely *N*. *winkelhorsti* because the humeri are not pachyostotic as should be the case for *Lariosaurus*. The rest of the humeri from Winterswijk might belong to *N*. *marchicus*, but show morphological, histological, and microanatomical variability. The presence of a third *Nothosaurus* species at this locality is also conceivable [14,15]. SMNS 54317 represents an early ontogenetic stage based on bone tissue and vascularization. This sample and MB.R. 782, however, fit the histological and microanatomical variability of what is thought to represent *N*. *marchicus*. MB.R. 817.1 and MB.R. 780 from the Lower Muschelkalk of Upper Silesia are both much larger than humeri from Winterswijk (Table 1). They differ distinctly in microanatomy (and morphology) when compared to each other as well as to the Winterswijk sample, most likely representing two different taxa, one of which may be *Germanosaurus*. Rieppel and Wild [9] noted that *Nothosaurus mirabilis* may extend down into the early Anisian (upper Lower Muschelkalk; mu2; [9]).

According to Rieppel and Wild [9] and Rieppel [1], a small-bodied nothosaur (cf. *N*. *marchicus*) as well as a larger-bodied nothosaur (cf. *N*. *mirabilis*) occur in the Middle Muschelkalk. Klein [12] described humeri from the Middle Muschelkalk of Freyburg (River Unstrut), which differs in morphology, microanatomy, and histology from *N*. *marchicus* and might represent *N*. *mirabilis*. However, sexual dimorphism for both taxa cannot be excluded as an explanation of these histological and microanatomical differences.

Except for a tendency towards a larger medullary cavity, samples IGWH-25 and IGWH-28 are morphologically and histologically (bone tissue and vascularization) similar to what is thought to represent *N*. *marchicus*. The microanatomy and bone tissue of MB.R. 162.4, MB.R. 477, and MB.R. 174.2 are comparable to *N*. *marchicus* but are morphologically different. Again, morphological differences might be related to developmental plasticity or sexual dimorphism. Histology and microanatomy of MHI 1193 is similar to the Lower Muschelkalk samples SMNS 80154 and Wijk 13–259 and might indicate the presence of diminutive taxon in the Middle Muschelkalk. Most of the Middle Muschelkalk samples are larger than the known size range of *N*. *marchicus* and have a tendency towards a decrease in bone mass. These humeri differ also morphologically from *N*. *marchicus* and have been assigned before to another humeral morphotype of unknown affinity [12]. Sample MB.R. 414 with its spongy periosteal tissue clearly differs from all other Middle Muschelkalk samples and may indicate the presence of yet another nothosaur taxon if other explanations can be excluded. MB.R 539 from the Middle Muschelkalk of Rüdersdorf has a very distinct cross section, with a characteristically pointed preaxial margin and a broad but also pointed postaxial margin. Its bone tissue is characterized by an untypical high amount of lamellar bone. Its overall humeral morphology, however, is that of a typical *Nothosaurus*.

Small humeri collected from the Upper Muschelkalk of Crailsheim (GPIT/RE/sample, Table 1) are unified by morphology, histology, and microanatomy that clearly differ from those in *N*. *marchicus*. The morphology of these humeri differs also from that of *N*. *jagisteus* [10]. No humeri are available for comparison for *N*. *edingerae* and *N*. *juvenilis*, the other small-bodied nothosaur taxa described from the Upper Muschelkalk (see S1 Text and S1 Table) [1,10]. Early ontogenetic stages of larger-bodied nothosaur taxa can be excluded for these smaller Upper Muschelkalk specimens due to morphology and histology. The morphology of humerus SMNS 53012 is obscured by compression, but histology and microanatomy fit well with the above mentioned GPIT/RE samples indicating similar taxonomical affinities.

SMNS 2557 most likely represents a juvenile of the larger-bodied nothosaur exhibiting BMI (SMNS 84851, SMNS 7175) due to its small size, the absence of a free medullary cavity, and the presence of calcified cartilage at midshaft. The majority of large humeri from the Upper Muschelkalk show some degree of BMD (Table 1). These most likely belong either to *N*. *mirabilis* or to *N*. *giganteus*. As already noted, *Simosaurus* can be excluded from consideration due to different morphology, microanatomy, and histology [16]. Thus, either *N*. *mirabilis* or *N*. *giganteus* show in general BMI or BMD, respectively. The taxonomic assignment of StIPB R 40, StIPB R 45, and StIPB R 53 by Krahl et al. [24] to *N*. *giganteus* and of StIPB R 54/2 to *N*. *mirabilis* was solely based on the size of the humeri and cannot be verified. The microanatomy of these samples is very similar and taxonomic distinction based on bone histology is not possible.

*N*. *mirabilis* and *N*. *giganteus* differ in the length of their neural spines [9] which is also related to swimming style. *N*. *giganteus* has short neural spines, which is a plesiomorphic feature for *Nothosaurus*, whereas *N*. *mirabilis* has elongated neural spines [9]. *N*. *marchicus* has short neural spines and its humerus is osteosclerotic. In *N*. *jagisteus* the length of the neural spines is intermediate but microanatomy of the humerus is not known [10]. Based on these differences, we speculate that *N*. *giganteus* is represented by the plesiomorphic histological pattern of retaining BMI, in keeping with its short neural spines. *N*. *mirabilis* could be represented in our sample by the humeri with the very thin cortex, uniting the derived states of BMD and high neural spines, reflecting evolution towards an active swimming style.

## Conclusions

The four major groups of Triassic Sauropterygia (Placodontia, Pachypleurosauria, Nothosauroidea, Pistosauroidea) show a general tendency for osteosclerosis and bone mass increase [12,16,23–25,30,31,36,39–41]. However, among *Nothosaurus* spp. from the latest Anisian and early Ladinian (Middle Triassic; Upper Muschelkalk deposits) occur some large-bodied individuals (or taxa) that are unique among Sauropterygia in documenting a distinct decrease in bone mass. This has resulted in a very thin-walled cortex in some individuals. Another peculiar pattern observed in some other individuals of *Nothosaurus* from the latest Anisian and early Ladinian is a heterogeneously spongious periosteal cortex. Both patterns are unique among amniotes. The difference to the thin-walled bones of birds is that the former are pneumatized, which certainly was not the case for nothosaurs.

Quantitative analyses reveal that size, indicated by the midshaft width proxy, has an influence on bone compactness. Smaller taxa have generally higher bone compactness than larger taxa throughout the evolutionary history of the group. They also showed that there is a significant difference between the four microanatomical categories documented among the nothosaur sample, and also that there is a significant difference in the distribution of the quantitative microanatomical parameters following the stratigraphy (i.e., between specimens from the Lower, Middle and Upper Muschelkalk, respectively) reflecting specificities linked to each stratigraphic level, such as that Lower Muschelkalk specimens generally all show a compact organization, whereas Middle Muschelkalk and Upper Muschelkalk specimens show a high diversity in the types of patterns observed.

Small bodied taxa with their compact organization are assumed to have been bound throughout their evolution to near coastal environments, whereas large bodied *Nothosaurus* spp. diversified from the middle Anisian onwards and settled near coastal environments as well as in open water. Microanatomical diversity observed in *Nothosaurus* spp. may reflect developmental plasticity, sexual dimorphism, but also taxonomical diversity.

## Supporting Information

S1 Table Table on distribution of *Nothosaurus* taxa in the Muschelkalk and Keuper (early Anisian- early Ladinian) of the Germanic Basin including localities of which samples are included into the current study. (DOC)

S2 Table Bone profiler parameters. **C** is the global bone compactness for the whole sectional area. **S** is the reciprocal of the slope at the inflection point and generally reflects the width of the transition zone between the cortical bone and the medullary region. **P** is the relative distance from the center of the section to the point of inflection, i.e. where the most abrupt change in compactness is observed. P is thus proportional to the size of the medullary cavity. **C** is the global bone compactness for the whole sectional area. **S** is the reciprocal of the slope at the inflection point and generally reflects the width of the transition zone between the cortical bone and the medullary region. **P** is the relative distance from the center of the section to the point of inflection, i.e., where the most abrupt change in compactness is observed. P is thus proportional to the size of the medullary cavity.
(DOC)

S1 Text Sea level and distribution of *Nothosaurus* spp. in the Germanic Basin (Muschelkalk and Keuper deposits) during the Anisian and Ladinian. (DOC)

## References

[1] Rieppel O. Sauropterygia I—Placodontia, Pachypleurosauria, Nothosauroidea, Pistosauroidea In: Wellnhofer P, editor. Handbuch der Paläoherpetologie. München Verlag Dr. Friedrich Pfeil 2000; 12A:1–134.

[2] Motani R. The evolution of marine reptiles. Evolution: Education and Outreach. 2009;2:224–35 [10.1007/s12052-009-0139-y].

[3] Hänni K. Die Gattung *Ceresiosaurus*. *Ceresiosaurus calgagnii* Peyer und *Ceresiosaurus lanzi* n.sp. (Lariosauridae, Sauropterygia) Zürich: vdf Hochschulverlag AG; 2004. 146 p.

[4] Hvon Meyer. *Simosaurus*, die Stumpfschnauze, ein Saurier aus dem Muschelkalke von Luneville. Neues Jahrbuch für Mineralogie, Geognosie, Geologie und Petrefaktenkunde. 1842;1842:184–98.

[5] Rieppel O. Osteology of *Simosaurus gaillardoti* and the relationships of stem-group Sauropterygia. Fieldiana (Geology), New Series. 1994;28:1–85.

[6] Tschanz K. *Lariosaurus buzzii* n. sp. from the Middle Triassic of Monte San Giorgio (Switzerland) with comments on the classification of nothosaurs. Palaeontographica Abt A. 1989;208(4–6):153–79.

[7] Rieppel O. Feeding mechanics in Triassic stem-group sauropterygians: the anatomy of a successful invasion of Mesozoic seas. Zoological Journal of the Linnean Society. 2002;135(1):33–63.

[8] Hvon Meyer. Die Saurier des Muschelkalks mit Rücksicht auf die Saurier aus Buntem Sandstein und Keuper Zur Fauna der Vorwelt, zweite Abtheilung, VIII+ 167 pages. Frankfurt: Heinrich Keller; 1847–1855.

[9] Rieppel O, Wild R. A revision of the genus *Nothosaurus* (Reptilia: Sauropterygia) from the Germanic Triassic, with comments on the status of *Conchiosaurus clavatus*. Fieldiana (Geology), New Series. 1996;No. 34:1–82.

[10] Rieppel O. A new species of *Nothosaurus* (Reptilia: Sauropterygia) from the Upper Muschelkalk (Lower Ladinian) of southwestern Germany. Palaeontographica, Abt A. 2001;263(1–6):137–61.

[11] Sues H-D. The postcranial skeleton of *Pistosaurus* and the interrelationships of the Sauropterygia (Diapsida). Zoological Journal of the Linnean Society. 1987;90(2):109–31.

[12] Klein N. Long bone histology of Sauropterygia from the Lower Muschelkalk of the Germanic Basin provides unexpected implications for phylogeny. PLoS ONE. 2010;5(7): e11613 10.1371/journal.pone.0011613 20657768 PMC2908119

[13] Bickelmann C, Sander PM. A partial skeleton and isolated humeri of *Nothosaurus* (Reptilia: Eosauropterygia) from Winterswijk, The Netherlands. Journal of Vertebrate Paleontology. 2008;28(2):326–38 [10.1671/0272-4634(2008)28[326:APSAIH]2.0.CO;2].

[14] Klein N, Voeten DFAE, Lankamp J, Bleeker R, Sichelschmidt OJ, Liebrand M, et al Postcranial material of *Nothosaurus marchicus* from the Lower Muschelkalk (Anisian) of Winterswijk, The Netherlands, with remarks on swimming styles and taphonomy. Paläontologische Zeitschrift. 2015,89(4):961–981.

[15] Voeten DFAE, Sander PM, Klein N. Skeletal material from larger Eusauropterygia (Reptilia: Eosauropterygia) with nothosaurian and cymatosaurian affinities from the Lower Muschelkalk of Winterswijk, The Netherlands. Paläontologische Zeitschrift. 2015,89 (4):943–960.

[16] Klein N, Griebeler EM. Bone histology, microanatomy, and growth of the nothosauroid *Simosaurus gaillardoti* (Sauropterygia) from the Upper Muschelkalk of southern Germany/Baden-Württemberg. Comptes Rendus Palevol. 2016,15(1–2):142–162.

[17] Braun J, Reif W-E. A survey of aquatic locomotion in fishes and tetrapods. Neues Jahrbuch für Geologie und Paläontologie, Abhandlungen. 1985;169:307–312.

[18] Watson DMS. The elasmosaurid shoulder-girdle and fore-limb. Proceedings of the Zoological Society London. 1924;24:885–917.

[19] Fvon Huene. Ein beachtenswerter Humerus aus unterstem Muschelkalk und seine Bedeutung. Neues Jahrbuch für Mineralogie, Monatshefte Abt B. 1944;1944(7/9):223–7.

[20] Carroll RL, Gaskill P. The nothosaur *Pachypleurosaurus* and the origin of plesiosaurs. Philosophical Transactions of the Royal Society of London Series B. 1985;309(1139):343–93.

[21] Feldkamp SD. Foreflipper propulsion in the California sea lion, *Zalophus californianus*. Journal of Zoology. 1987;212:43–57.

[22] Zhang Q, Wen W, Hu S, Benton MJ, Zhou C, Xie T, et al Nothosaur foraging tracks from the Middle Triassic of southwestern China. Nature Communications. 2014;5:3973 [10.1038/ncomms4973]. 24917514

[23] Hugi J. The long bone histology of *Ceresiosaurus* (Sauropterygia, Reptilia) in comparison to other eosauropterygians from the Middle Triassic of Monte San Giorgio (Switzerland/Italy). Swiss Journal of Palaeontology. 2011;130(2):297–306 [10.1007/s13358-011-0023-6].

[24] Krahl A, Klein N, Sander PM. Evolutionary implications of the divergent long bone histologies of *Nothosaurus* and *Pistosaurus* (Sauropterygia, Triassic). BMC Evolutionary Biology. 2013;13:1–23 [10.1186/1471-2148-13-123]. 23773234 PMC3694513

[25] Klein N, Houssaye A, Neenan JM, Scheyer TM. Long bone histology and microanatomy of Placodontia (Diapsida: Sauropterygia). Contributions to Zoology. 2015b;84(1):59–84.

[26] Peyer B. Die Triasfauna der Tessiner Kalkalpen XIV. *Paranothosaurus amsleri* nov. gen. nov. spec. Abhandlungen der Schweizerischen Paläontologischen Gesellschaft. 1939;62:1–87.

[27] Klein N, Sander PM. Bone histology and growth of the prosauropod dinosaur *Plateosaurus* from the Norian bonebeds of Trossingen (Germany) and Frick (Switzerland). Special papers in Paleontology.2007; 77:169–206.

[28] Francillon-Vieillot H, Buffrénil Vde, Castanet J, Géraudie J, Meunier FJ, Sire, et al Microstructure and mineralization of vertebrate skeletal tissues In: Carter JG, editor. Skeletal Biomineralization: Patterns, Processes and Evolutionary Trends. Vol. 1 New York: Van Nostrand Reinhold; 1990 p. 471–530.

[29] Girondot M, Laurin M. Bone profiler: a tool to quantify, model, and statistically compare bone-section compactness profiles. Journal of Vertebrate Paleontology. 2003;23(2):458–61 [10.1671/0272-4634(2003)023[0458:BPATTQ]2.0.CO;2].

[30] Klein N. Postcranial morphology and growth of the pachypleurosaur *Anarosaurus heterodontus* (Sauropterygia) from the Lower Muschelkalk of Winterswijk, The Netherlands. Paläontologische Zeitschrift. 2012;86(4):389–408 [10.1007/s12542-012-0137-1].

[31] Hugi J, Scheyer TM, Sander PM, Klein N, Sánchez-Villagra MR. Long bone microstructure gives new insights into the life of pachypleurosaurids from the Middle Triassic of Monte San Giorgio, Switzerland/Italy. Comptes Rendus Palevol. 2011;10(5–6):413–26 [10.1016/j.crpv.2011.03.009].

[32] Klein N, Neenan JM, Scheyer TM, Griebeler E-M. Growth patterns and life history strategies in Placodontia (Diapsida: Sauropterygia). Royal Society Open Science. 2015c;2:140440 [10.1098/rsos.140440]. 26587259 PMC4632572

[33] Klein N, Sander PM. Ontogenetic stages in the long bone histology of sauropod dinosaurs. Paleobiology. 2008;34(2):247–63 [10.1666/0094-8373(2008)034[0247:OSITLB]2.0.CO;2].

[34] Sander PM. Life history of Tendaguru sauropods as inferred from long bone histology. Mitteilungen Museum für Naturkunde Berlin, Geowissenschaftliche Reihe. 1999;2:103–12.

[35] Canoville A, Laurin M. Evolution of humeral microanatomy and lifestyle in amniotes, and some comments on palaeobiological inferences. Biological Journal of the Linnean Society. 2010;100(2):384–406 [10.1111/j.095-8312.2010.01431.x].

[36] Sander PM. Skeletochronology in the small Triassic reptile *Neusticosaurus*. Annales des Sciences Naturelles, Zoologie. 1990;11:213–7.

[37] Digby KH. The measurements of diaphyseal growth in the proximal and distal directions. Journal of Anatomy and Physiology. 1915;50:187–8. PMC1289069 17233059

[38] Gray DJ, Gardner E. The prenatal development of the human humerus. The American Journal of Anatomy. 1969;124:431–45. 5774654 10.1002/aja.1001240403

[39] Ade Ricqlès, Vde Buffrénil. Bone histology, heterochronies and the return of tetrapods to life in water: where are we? In: Mazin J-M, Buffrénil Vde, editors. Secondary Adaptations of Tetrapods to Life in Water. München: Verlag Dr. Friedrich Pfeil; 2001 p. 289–310.

[40] Vde Buffrénil, Mazin J-M. Contribution de l’histologie osseuse à l’interprétation paléobiologique du genre *Placodus* Agassiz, 1833 (Reptilia, Placodontia). Revue de Paléobiologie. 1992;11(2):397–407.

[41] Wiffen J, Buffrénil Vde, Ricqlès Ade, Mazin J-M. Ontogenetic evolution of bone structure in Late Cretaceous Plesiosauria from New Zealand. Geobios. 1995;28(5):625–40.

[42] Konietzko-Meier D, Schmitt A. A histological study of a femur of *Plagiosuchus*, a Middle Triassic temnospondyl amphibian from southern Germany, using thin sections and micro-CT scanning. in: Mulder EWA, Jagt JWM, Schulp AS, editors. The Sunday’s child of Dutch earth sciences—a tribute to Bert Boekschoten on the occasion of his 80th birthday. Netherlands Journal of Geosciences. 2013;92(2–3):97–108.

[43] Houssaye A, Lindgren J, Pellegrini R, Lee AH, Germain D, Polcyn MJ. Microanatomical and histological features in the long bones of mosasaurine mosasaurs (Reptilia, Squamata)–implications for aquatic adaptation and growth rates. PLoS ONE. 2013;8(10): e76741 [10.1371/journal.pone.0076741]. 24146919 PMC3797777

[44] Houssaye A, Scheyer TM, Kolb C, Fischer V, Sander PM. A new look at ichthyosaur long bone microanatomy and histology: Implications for their adaptation to an aquatic life. PLoS ONE. 2014;9(4): e95637 10.1371/journal.pone.0095637 24752508 PMC3994080

[45] Shelton CD, Sander PM, Stein K, Winkelhorst H. Long bone histology indicates sympatric species of *Dimetrodon* (Lower Permian, Sphenacodontidae). Earth and Environmental Science Transactions of the Royal Society of Edinburgh. 2013;103:217–36 [10.1017/S175569101300025X].

[46] Laurin M, Buffrénil de V. Microstructural features of the femur in early ophiacodontids: A reappraisal of ancestral habitat use and lifestyle of amniotes. Comptes Rendus Palevol. 2016;15 (1–2):115–127.

[47] Houssaye A. "Pachyostosis" in aquatic amniotes: a review. Integrative Zoology. 2009;4(4):325–40 [10.1111/j.749-4877.2009.00146.x]. 21392306

[48] Gray N-M, Kainec K, Madar S, Tomko L, Wolfe S. Sink or swim? Bone density as a mechanism for buoyancy control in early cetaceans. The Anatomical Record. 2007;290:638–53 [10.1002/ar.20533]. 17516430

[49] Vde Buffrénil, Canoville A, D'Anastasio R, Domning DP. Evolution of sirenian pachyosteosclerosis, a model-case for the study of bone structure in aquatic tetrapods. Journal of Mammalian Evolution. 2010;17(2):101–20 [10.1007/s10914-010-9130-1].

[50] Houssaye A. Bone histology of aquatic reptiles: what does it tell us about secondary adaptation to an aquatic life? Biological Journal of the Linnean Society. 2013;108(1):3–21 [10.1111/j.095-8312.2012.02002.x].

[51] Laurin M, Canoville A, Germain D. Bone microanatomy and lifestyle: a descriptive approach. Comptes Rendus Palevol. 2011;10(5–6):381–402 [10.1016/j.crpv.2011.02.003].

[52] Hayashi S, Houssaye A, Nakajima Y, Chiba K, Ando T, Sawamura H, et al Bone inner structure suggests increasing aquatic adaptations in Desmostylia (Mammalia, Afrotheria). PLoS ONE. 2013;8(4):e59146 10.1371/journal.pone.0059146 23565143 PMC3615000

[53] Houssaye A, Waskow K, Hayashi S, Cornette R, Lee AH, Hutchinson J. Biomechanical evolution of solid bones in large animals: a microanatomical investigation. Biological Journal of the Linnean Society. 2015; [10.1111/bij.12660].

[54] Vde Buffrénil. Donnés préliminaires sur la structure des marques de croissance squelettiques chez les crocodiliens actuels et fossiles [Preliminary data on the structure of growth marks among living and fossil crocodilians]. Bulletin de la Societé de Zoologie de France. 1980;105:355–61.

[55] Klein N, Scheyer T, Tütken T. Skeletochronology and isotopic analysis of a captive individual of *Alligator mississippiensis* Daudin, 1802. Fossil Record. 2009;12(2):121–31 [10.1002/mmng.200900002].

[56] Houssaye A. Palaeoecological and morphofunctional interpretation of bone mass increase: an example in Late Cretaceous shallow marine squamates. Biological Review (2013), 88, pp. 117–139. 10.1111/j.1469-185X.2012.00243.x 22943660

[57] Buffrénil Vde, Castanet J. Age estimation by skeletochronology in the Nile monitor (*Varanus niloticus*), a highly exploited species. Journal of Herpetology. 2000;34:414–24.

[58] Buffrénil Vde, Houssaye A, Böhme W. Bone vascular supply in monitor lizards (Squamata: Varanidae): influence of size, growth, and phylogeny. Journal of Morphology. 2008;269:533–43. 18157866 10.1002/jmor.10604

[59] Hugi J, Sánchez-Villagra MR. Life history and skeletal adaptations in the Galapagos marine iguana (*Amblyrhynchus cristatus*) as reconstructed with bone histological data—a comparative study of iguanines. Journal of Morphology. 2012;46(3):312–24 [10.1670/11-071].

[60] Klein N, Albers PCH. A new species of the sauropsid reptile *Nothosaurus* from the Lower Muschelkalk of the western Germanic Basin, Winterswijk, The Netherlands. Acta Palaeontologica Polonica. 2009;54(4):589–98 [10.4202/app.2008.0083].

[61] Albers PCH. New *Nothosaurus* skulls from the Lower Muschelkalk of the western Lower Saxony Basin (Winterswijk, The Netherlands) shed new light on the status of *Nothosaurus winterswijkensis*. Netherlands Journal of Geosciences—Geologie en Mijnbouw. 2011;90(1):15–22.

[62] Klein N, Voeten DFAE, Haarhuis A, Bleeker R. The earliest record of the genus *Lariosaurus* from the early middle Anisian (Middle Triassic) of the Germanic Basin. Journal of Vertebrate Paleontology 2016; 10.1080/02724634.2016.1163712

