# Supplementary material for: Diverse Aquatic Adaptations in Nothosaurus spp. (Sauropterygia)—Inferences from Humeral Histology and Microanatomy
Source: PLoS One. 2016 Jul 8;11(7):e0158448. doi: 10.1371/journal.pone.0158448 (PMC4938232; doi:10.1371/journal.pone.0158448)
Supplement: S1 Table — (DOC) [file pone.0158448.s001.doc]

**S1 Table. Table on distribution of *Nothosaurus* taxa in the Muschelkalk and Keuper (early Anisian- early Ladinian) of the Germanic Basin including localities of which samples are included into the current study.**

| **Locality** | **Stratigraphy** | ***Nothosaurus* Taxa** | **Fazies/**  **Environment** | **sampled specimens** |
| --- | --- | --- | --- | --- |
| **Lower Muschelkalk** |  |  |  |  |
| Górny Śląsk  (formerly Upper Silesia, Poland | late early Anisian | cf. *N. marchicus* | shallow marine-, carbonate ramp-, and near coastal environments | MB.R. 782, MB.R. 780, MB.R. 817.1 |
| Winterswijk  (The Netherlands) | late early Anisian | *N. marchicus* | coastal environment; carbonate tidal flats | Wijk13-89, Wijk05-9, Wijk12-91, Wijk13-259, Wijk11-87, Wijk10-170, Wijk11-265, Wijk13-141, Wijk12-91, Wijk11-20 |
| Eschenbach in der Oberpfalz (Bavaria, Germany) | late early Anisian | *Nothosaurus* sp. | ? | SMNS 54317 |
| **Middle Muschelkalk** |  |  |  |  |
| Freyburg on the river Unstrut (Saxony, Germany), Rüdersdorf  (near Berlin, Germany), Oberdorla and Jena (both Thuringia, Germany), Förderstedt by Magdeburg (Germany) | early middle Anisian | cf. *N. marchicus* | fully marine;  center of basin  but regression has started | IGWH 4, IGWH 17, IGWH 18, IGWH 7, IGWH 14, IGWH 8, IGWH 25, IGWH 28, IGWH 3, MB.R. 174.2, MB.R. 477, MB.R. 162.4 MB.R. 414, MB.R. 539, MB.R. 941 |
| **Upper Muschelkalk** |  |  |  |  |
| Gaismühle/Crailsheim | mo1  (middle Anisian) | *Nothosaurus* sp. |  | SMNS 17214 |
| Wiesloch near Heidelberg | mo1  (middle Anisian) | *N. juvenilis* |  | no sample |
| Bayreuth |  | *Nothosaurus* sp. |  | MB.R.270, MB.R.272, MB.R.278, MB.R.279, MB.R.281, MB.R.282, |
| Bayreuth/Laineck | mo1  (middle Anisian) | *N. mirabilis*  *N. giganteus* |  | StIPB R 40, StIPB R 45, StIPB R 53, StIPB R 54, PIMUZ AIII-1, PIMUZ AIII-2 |
| Bindlach/Bayreuth | mo2  (late Anisian) | *N. mirabilis* |  | SMNS 84772 |
| Gundelsheim/Neckar | mo2  (late Anisian) | *N. giganteus* |  | SMNS 81988 (laut Wild ?*mirabilis*) |
| Künzelsau | mo2  (late Anisian) | *Nothosaurus* sp. |  | MHI 873 |
| Scheuerbach | ?mo2/mo3 (Ladinian) |  |  | MHI 1906 |
| Cassina, Meride, canton Ticino, Switzerland | early Ladinian | *Ceresiosaurus lanzi* |  | PIMUZ 4845 |
| Berlichingen/Hohenlohe | mo3  (Ladinian) | *N. mirabilis*  *N. jagisteus* |  | no sample |
| Crailsheim |  | *Nothosaurus* sp. |  | GPIT/RE/1590a, GPIT/RE/1590b, GPIT/RE/1590d,  GPIT/RE/1339a, GPIT/RE/1339b, GPIT/RE/1339d, GPIT/RE/1339f |
| Tiefenbach/Crailsheim | ?mo3 (Ladinian) | *N. giganteus* |  | SMNS 84851 |
| Heldenmühle/Crailsheim | ?mo3  (Ladinian) | *N. mirabilis*  *N.* *giganteus* |  | SMNS 17882 |
| Steinbruch Rüblingen | mo3 (Ladinian) | *N. giganteus* |  | SMNS 53012 (?young), SMNS 50221 |
| Schmalfelden | mo3 (Ladinian) | *N. giganteus* |  | MHI 633 |
| Wilhelmsglück/Schwäbisch-Hall | mo3 (Ladinian) | *Nothosaurus* sp. |  | MHI 1978 |
| Barenhaldenmühle/  Satteldorf/Crailheim | ku (Ladinian)  Grenzbonebed | *Nothosaurus* sp. |  | MHI 754 |
| Hoheneck near Ludwigsburg  *Neusticosaurus* !!! | ku (Ladinian) | *N. mirabilis*  *N. giganteus* | marine-brackish | SMNS 2557 (small taxon), MB.R.269, SMNS 7175 |
| Herdlingshagen | ku (Ladinian) | *Nothosaurus* sp. | marine-brackish | SMNS 80688 |
| Rotmaintal near Bayreuth | middle ku  (Carnian) | *N. edingerae* |  | no sample |
