# Supplementary material for: Diverse Aquatic Adaptations in Nothosaurus spp. (Sauropterygia)—Inferences from Humeral Histology and Microanatomy
Source: PLoS One. 2016 Jul 8;11(7):e0158448. doi: 10.1371/journal.pone.0158448 (PMC4938232; doi:10.1371/journal.pone.0158448)
Supplement: S2 Table — C is the global bone compactness for the whole sectional area. S is the reciprocal of the slope at the inflection point and generally reflects the width of the transition zone between the cortical bone and the medullary region. P is the relative distance from the center of the section to the point of inflection, i.e. where the most abrupt change in compactness is observed. P is thus proportional to the size of the medullary cavity. C is the global bone compactness for the whole sectional area. S is the reciprocal of the slope at the inflection point and generally reflects the width of the transition zone between the cortical bone and the medullary region. P is the relative distance from the center of the section to the point of inflection, i.e., where the most abrupt change in compactness is observed. P is thus proportional to the size of the medullary cavity. (DOC) [file pone.0158448.s002.doc]

**S2 Table. Bone profiler parameters.**

| **Specimen** | **C** | **S** | **P** |
| --- | --- | --- | --- |
| Wijk11-87 | 0,856 | 0,107 | 0,240 |
| Wijk13-89 | 0,916 | 0,034 | 0,137 |
| Wijk13-141 | 0,867 | 0,086 | 0,251 |
| Wijk11-265 | 0,875 | 0,019 | 0,277 |
| Wijk12-91 | 0,887 | 0,102 | 0,195 |
| Wijk11-20 | 0,797 | 0,046 | 0,347 |
| MB.R. 780 | 0,914 | 0,113 | 0,897 |
| IGWH 3 | 0,814 | 0,054 | 0,350 |
| IGWH 25 | 0,751 | 0,076 | 0,422 |
| MB.R. 174-2 | 0,874 | 0,179 | 0,130 |
| IGWH 14 | 0,492 | 0,079 | 0,625 |
| IGWH 7 | 0,613 | 0,037 | 0,582 |
| IGWH 18 | 0,701 | 0,081 | 0,642 |
| IGWH 8 | 0,653 | 0,178 | 0,519 |
| MB.R. 414 | 0,711 | 0,058 | 0,319 |
| IGWH 4 | 0,745 | 0,058 | 0,629 |
| MB.R. 539 | 0,738 | 0,068 | 0,465 |
| MB.R. 941 | 0,638 | 0,079 | 0,768 |
| GPIT 1590d | 0,884 | 0,187 | 0,054 |
| GPIT 1339b | 0,874 | 0,025 | 0,388 |
| SMNS 53012 | 0,716 | 0,064 | 0,427 |
| GPIT 1339f | 0,911 | 0,243 | 0,001 |
| GPIT 1590b | 0,913 | 0,054 | 0,120 |
| GPIT 1339d | 0,936 | 0,043 | 0,001 |
| SMNS 2557 | 0,931 | 0,094 | 0,001 |
| MHI 633 | 0,827 | 0,046 | 0,267 |
| SMNS 50221 | 0,796 | 0,115 | 0,748 |
| SMNS 17214 | 0,583 | 0,125 | 0,618 |
| SMNS 84772 | 0,725 | 0,202 | 0,285 |
| MHI 754 | 0,709 | 0,088 | 0,444 |
| MHI 1978 | 0,876 | 0,063 | 0,227 |
| MB.R. 279 | 0,469 | 0,061 | 0,760 |
| MB.R. 282 | 0,782 | 0,005 | 0,023 |
| MB.R. 278 | 0,452 | 0,065 | 0,681 |
| MB.R. 281 | 0,688 | 0,079 | 0,530 |
| MHI 873 | 0,363 | 0,110 | 0,810 |
| SMNS 7175 | 0,948 | 0,696 | 0,001 |
| MB.R. 272 | 0,383 | 0,134 | 0,786 |
| SMNS 81988 | 0,524 | 0,072 | 0,700 |
| MB.R. 270 | 0,713 | 0,094 | 0,526 |
| MB.R. 269 | 0,501 | 0,081 | 0,824 |
| StIPB R54/2 | 0,571 | 0,127 | 0,642 |
| StIPB R 45 | 0,510 | 0,094 | 0,714 |
| StIPB R 53 | 0,216 | 0,017 | 0,815 |
| StIPB R 40 | 0,365 | 0,083 | 0,801 |
| PIMUZ AIII-1 | 0,252 | 0,045 | 0,861 |
| PIMUZ AIII-2 | 0,374 | 0,107 | 0,805 |
| PIMUZ 4845 | 0,917 | 0,030 | 0,001 |

C is the global bone compactness for the whole sectional area. S is the reciprocal of the slope at the inflection point and generally reflects the width of the transition zone between the cortical bone and the medullary region. P is the relative distance from the center of the section to the point of inflection, i.e. where the most abrupt change in compactness is observed. P is thus proportional to the size of the medullary cavity.
