# Supplementary material for: Diverse Aquatic Adaptations in Nothosaurus spp. (Sauropterygia)—Inferences from Humeral Histology and Microanatomy
Source: PLoS One. 2016 Jul 8;11(7):e0158448. doi: 10.1371/journal.pone.0158448 (PMC4938232; doi:10.1371/journal.pone.0158448)
Supplement: S1 Text — (DOC) [file pone.0158448.s003.doc]

**S1 Text**

**Sea level and distribution of *Nothosaurus* spp. in the Germanic Basin (Muschelkalk and Keuper deposits) during the Anisian and Ladinian**

During the Anisian and Ladinian (Middle Triassic), the Germanic Basin was flooded by the Muschelkalk Sea and only had a few temporary open marine connections to the western Tethys [63]. The entire Muschelkalk is regarded as marine, but due to transgression and regression events, sea level varied both geographically and stratigraphically. If sea level was temporarily high, the Germanic Sea was nevertheless dominated by shallow marine and near-coastal marine reptiles. Pelagic taxa such as ichthyosaurs remain very rare and are interpreted as occasional or stray visitors.

The localities in the vicinity of Górny Śląsk (formerly Upper Silesia), Poland belong to the Gogolin Formation, which is interpreted as fully marine and yielded one of the oldest vertebrate faunas within the Germanic Basin [64]. The sediments represent shallow marine, carbonate ramp, and near coastal environments. In Germany, the Jena Formation (including the Wellenkalk facies), which is slightly younger than the Gogolin Formation, was deposited during the Lower Muschelkalk (early Anisian), and sediments represent the center of the basin that was fully marine [63]. The Vossenveld Formation crops out at the locality of Winterswijk, which is early Anisian in age, too and correlates with the Wellenkalk facies in Germany [65]. Sediments in Winterswijk indicate periodical subaerial exposure and drying of the surface. At other times, the area was covered by shallow water. This indicates a coastal environment similar to modern days carbonate tidal flats [66,67]. Klein et al. [14] hypothesized that skeletons/bodies of larger marine reptiles were brought in from further offshore by water currents.

From the Lower Muschelkalk of Upper Silesia, the nothosaur *Germanosaurus* is described on the basis of two skulls [1]. The presence of *N. marchicus* is assumed due to numerous nothosaur bones [9]. Several dozens of nothosaur skulls are available from various localities from central Germany and have been assigned to *N. marchicus*, but they show a high variability in morphological features [1,9,12,60]. From Winterswijk *N. marchicus* is described based on skulls and articulated or associated postcranial material [14,14]. Humeral morphotype II of Bickelmann and Sander [13] can be linked to *N. marchicus* [12-14]. However, morphology and size range of Lower Muschelkalk nothosaur humeri and other postcranial bones are highly variable, leading to the hypotheses of: 1) a larger size range for *N. marchicus*, which has had a body length of around 1 to 1.5 m (see [14]) a possible sexual dimorphism for *N. marchicus* (expressed in size differences). Or 3) the presence of additional nothosaur taxa [12-14]. The diminutive *N. winkelhorsti* (skull length is 46 mm) is so far only known from Winterswijk (The Netherlands) and is based on one isolated skull. Recently, *Lariosaurus*, the sister taxon of *Nothosaurus* ([1] but see [68]) has been described from the Lower Muschelkalk of the Germanic Basin as well [62]. The postrcranial anatomy of *Lariosaurus* is very similar to that of *Nothosaurus* and the so far known specimens fall into the size range of *N. marchicus*. According to Rieppel and Wild [9] it is possible that *N. mirabilis* could reach back to the upper Lower Muschelkalk but evidence for this is based on a single skull. The upper part of the Jena Formation (Schaumkalk facies) occurs at the localities Freyburg on the river Unstrut (Saxony, Germany), Rüdersdorf (near Berlin, Germany), Oberdorla and Jena, (both Thuringia, Germany), and Förderstedt near Magdeburg (Germany). The sediments represent a sea level highstand before later dropping in the Middle Muschelkalk due to a regression phase associated with the temporary closure of the connections to the Tethys [63]. The dolomitic marls of the *orbicularis* beds (lowermost Middle Muschelkalk) in Rüdersdorf locality have yielded abundant nothosaur material [64] including a nearly complete skeleton of *N. marchicus* [9,69]. The Freyburg fauna has yielded a variety of isolated material of nothosaurs [63], including several nothosaur humeri [12]. cf. *N. marchicus* and a large nothosaur different from *N. mirabilis* are so far described based on morphology [9].

The Upper Muschelkalk represents a phase of open marine conditions with continuous high sea levels as a result of a transgression phase and a connection to the Tethys via the Burgundy Gate. The Upper Muschelkalk can be divided into three subunits (mo1 and mo2 that correspond to the Anisian); mo3, (Ladinian); e.g., [70]. Dozens of localities in southwest Germany have produced hundreds, if not thousands, of isolated nothosaur bones. The mo1 marks the beginning of the transgression, the mo2 represents the maximal transgression, and during the mo3 the sea level dropped again [71]. The peak of the transgression phase (mo2) is accompanied by an increase in faunal diversity, which is also reflected in an increase in taxonomical diversity of *Nothosaurus* [1,63].

In southwestern Germany, the boundary between Muschelkalk and Keuper (Ladinian) is defined by the base of the Grenzbonebed that is interpreted as a tempestitic condensation horizon [71]. The taxa of the Crailsheim Grenzbonebed vertebrate fauna are marine or limnic [71]. During the early late Ladinian (Lower Keuper) the Germanic Basin was influenced by changing sea levels and sedimentation rates that caused changing deltaic, limnic, and brackish conditions with short marine incursions.

Two large-bodied nothosaur taxa (*N. mirabilis*, skull length up to 460 mm, body length ~4 m; *N. giganteus*, skull length is up to 750 mm, body length ~6 m) lived contemporaneously among with two smaller-bodied taxa (i.e., *N. juvenilis,* skull length is 126 mm; *N. jagisteus*, skull length is 184 mm) in the Upper Muschelkalk of the Germanic Basin. In the Lower and Middle Keuper (upper Ladinian/lower Carnian) when marine influence was continuously decreasing small *N. edingerae* (skull length is 140 mm) is described but finds of larger-bodied nothosaurs are also still frequent. According to Rieppel and Wild [9] *N. marchicus* may occur up to the lower Upper Muschelkalk.

According to Peyer [26], the skull of a *N. giganteus* individual is 485 mm and the humerus 245 mm long, respectively. Some *N. giganteus* skulls must have been 1 m long indicated by fragmentary material. For *N. mirabilis* no comparable dimensions are available.

The humerus is articulated to a complete skeleton of *Ceresiosaurus* *lanzi* (PIMUZ T4845) originates from the Alpine Triassic (early Ladinian of Cassina, Meride, Canton Ticino, Switzerland), which represents shallow to open sea environments.

**References**

1. Hagdorn H, Rieppel O. Stratigraphy of marine reptiles in the Triassic of Central Europe. Zentralblatt für Geologie und Palaontologie. 1999: Teil I: 651–7678.
2. Hagdorn H, Rieppel O. Stratigraphy of marine reptiles in the Triassic of Central Europe. Zentralblatt für Geologie und Paläontologie. 1998; 7-8: 651–678.
3. Hagdorn H, Simon TH. Vossenveld-Formation. Litholex (Lithostratigraphische Einheiten Deutschlands).2010; ID 45: 1–6. http://www.bgr.de/app/litholex/gesamt_ausgabe_neu.php?id=45. 2010.
4. Oosterink HW. Winterswijk, Geologie Deel II. De Triasperiode

(geologie, mineralen en fossielen). Wetenschappelijke Mededelingen van de Koninklijke Nederlandse Natuurhistorische Vereniging. 1986; 178: 1–120.

1. Dülfer O, Klein N. Studentische Lehrgrabung im Winterswijker Muschelkalk. Der Präparator. 2006; 52: 90–96.
2. Liu J, Hu S-x, Rieppel O, Jiang D-y, Benton MJ, Kelley NP, et al. A gigantic nothosaur (Reptilia: Sauropterygia) from the Middle Triassic of SW China and its implication for the Triassic biotic recovery. Nature Scientific Reports. 2014;4: 7142 [doi: 10.1038/srep07142].
3. Schröder H. Wirbeltiere der Rüdersdorfer Trias. Abhandlungen der Königlich

Preussischen Geologischen Landesanstalt, Neue Folge. 1914;65:1–98.

1. Hagdorn H. The Muschelkalk in Germany. An Introduction. – In: Hagdorn H., editor Muschelkalk. A Field Guide. Korb (Goldschneck).1991; 9–21.
2. Hagdorn H, Reif WE. Die „Knochenbreccie von Crailsheim“ und weitere Mitteltrias-Bonebeds in Nordost-Württemberg – Alte und Neue Deutungen. In: Hagdorn, H., editor. Neue Forschungen zur Erdgeschichte von Crailsheim. Zur Erinnerung an Hofrat Richard Blezinger. (Sonderbände Ges. Naturk. Württemberg 1). Stuttgart (Goldschneck). 1988; 116–143.
